# De Novo Carcinoma after Solid Organ Transplantation to Give Insight into Carcinogenesis in General—A Systematic Review and Meta-Analysis

**DOI:** 10.3390/cancers13051122

**Published:** 2021-03-05

**Authors:** Eline S. Zwart, Esen Yüksel, Anne Pannekoek, Ralph de Vries, Reina E. Mebius, Geert Kazemier

**Affiliations:** 1 Amsterdam Universities Medical Centers, Cancer Center Amsterdam, Department of Surgery, VU University, 1081 HV Amsterdam, The Netherlands; e.zwart@amsterdamumc.nl (E.S.Z.); e.yuksel@amsterdamumc.nl (E.Y.); a.pannekoek@student.vu.nl (A.P.); 2 Amsterdam Universities Medical Centers, Department of Molecular Cell Biology and Immunology, VU University, 1081 HV Amsterdam, The Netherlands; r.mebius@amsterdamumc.nl; 3 Medical Library, Vrije Universiteit, 1081 HV Amsterdam, The Netherlands; r2.de.vries@vu.nl

**Keywords:** organ transplantation, carcinoma, epidemiologic studies, immunosuppression

## Abstract

**Simple Summary:**

Patients receiving a solid organ transplantation, such as a kidney, liver, or lung transplantation, inevitably have to take drugs to suppress the immune system in order to prevent rejection of the transplanted organ. However, these drugs are known to cause malignancies in the long term. This study focuses specifically on newly developed carcinomas in patients who use those drugs after a solid organ transplantation. This systematic review and meta-analysis of published data show a 20-fold risk to develop a carcinoma after solid organ transplantation compared to the general population, with specifically increased risks in patients who receive cyclosporine or azathioprine. By comparing the different pathways involved in immunosuppression and the occurrence of carcinoma development, new insights can be discovered for future research and understanding of carcinoma development in transplantation patients and the general population as well.

**Abstract:**

Immunosuppressive therapy after solid organ transplantation leads to the development of cancer in many recipients. Analysis of the occurrence of different types of de novo carcinomas in relation to specific immunosuppressive drugs may give insight into their carcinogenic process and carcinogenesis in general. Therefore, a systematic search was performed in Embase and PubMed. Studies describing over five de novo carcinomas in patients using immunosuppressive drugs after solid organ transplantation were included. Incidence per 1000 person-years was calculated with DerSimonian–Laird random effects model and odds ratio for developing carcinomas with the Mantel–Haenszel test. Following review of 5606 papers by title and abstract, a meta-analysis was conducted of 82 studies. The incidence rate of de novo carcinomas was 8.41. Patients receiving cyclosporine developed more de novo carcinomas compared to tacrolimus (OR1.56, 95%CI 1.00–2.44) and mycophenolate (OR1.26, 95%CI 1.03–1.56). Patients receiving azathioprine had higher odds to develop de novo carcinomas compared to mycophenolate (OR3.34, 95%CI 1.29–8.65) and head and neck carcinoma compared to tacrolimus (OR3.78, 95%CI 1.11–12.83). To conclude, patients receiving immunosuppressive drugs after solid organ transplantation have almost a 20-fold increased likelihood of developing carcinomas, with the highest likelihood for patients receiving cyclosporine A and azathioprine. Looking into altered immune pathways affected by immunosuppressive drugs might lead to better understanding of carcinogenesis in general.

## 1. Introduction

Solid organ transplantation patients receive different immunosuppressive drugs to prevent graft rejection. Each of these drugs inhibits the immune system in a specific manner. Calcineurin inhibitors, such as cyclosporin A (CsA) and tacrolimus (TAC), inhibit the proliferation of T cells which is important to prevent graft rejection [1,2]. Another group of immunosuppressive drugs, such as azathioprine (AZA) and mycophenolate (MMF), are called antimetabolites and inhibit DNA synthesis, thereby preventing proliferation of T and B cells [1]. Studies have demonstrated that MMF has a superior ability to prevent allograft rejection compared to AZA, which caused AZA to be mostly replaced by MMF [3,4,5,6]. Newer, more potent suppressors of lymphocyte proliferation, such as sirolimus (SIR) and everolimus (EVER), are inhibitors of the mammalian target of rapamycin (mTOR), which is an intracellular kinase involved in cell metabolism, growth, and proliferation (Table 1).

Even though outcomes of solid organ transplantation have improved dramatically since the discovery of immunosuppressive drugs, their use comes with a drawback. Overall, a two to seven times higher risk for development of de novo malignancies can be found in transplant recipients compared to the general population [7,8]. Long-term use of immunosuppressive agents is considered to be the major contributing factor [8]. Post-transplant lymphoproliferative disorders, (non-)melanoma skin cancer, and Kaposi’s sarcoma are among the most frequently occurring neoplasms after solid organ transplantation and they have been broadly investigated [9]. However, a large overview of occurrence of de novo carcinomas after solid organ transplantation is lacking. By analyzing the occurrence of different types of de novo carcinomas in relation to specific immunosuppressive drugs, insight can also be gained into the carcinogenesis process, providing new perspectives for translational cancer research. Therefore, the aim of this systematic review is to examine the overall and tumor-specific incidence of de novo carcinomas in varied solid organ transplant recipients using specific immunosuppressive drugs in order to gain insight into the pathways contributing to carcinogenesis in those patients, but also in the general population.

## 2. Materials and Methods

### 2.1. Search Strategy and Study Selection

This systematic review followed the Preferred Reporting Items for Systematic Reviews and Meta-Analyses (PRISMA) [10]. A literature search was conducted in the bibliographic databases of PubMed and Embase.com from inception up to September 10, 2020, in collaboration with a medical librarian. The following terms were used, including synonyms and closely related words, as index terms or free-text words: “Immunosuppression”, “Organ Transplantation”, and “Carcinoma”. The full search strategies can be found in Table S1. Title and abstracts were independently reviewed by E.Z. and A.P. After contemplation about conflicts, full texts were screened by A.P. and E.Y., and in case of conflict, E.Z. was consulted. All screening was conducted with the use of Rayyan, a systematic web app [11]. Studies that included solid organ transplant recipients of 18 years and older, who received chronic immunosuppression and developed a de novo carcinoma, were considered eligible. Studies written in languages other than English, literature reviews, studies describing less than five de novo carcinomas, studies describing recurrent hepatocellular carcinomas, studies describing premalignant lesions, and studies that did not describe the specific immunosuppressive treatment regimen were excluded. In case of overlapping databases, the study with the largest and most complete dataset was included.

### 2.2. Data Collection and Interpretation

Data of the included articles were extracted using a standardized data extraction form, including study design, patient demographics, duration of follow-up, number of transplant recipients, transplantation period, and number of patients with de novo carcinomas. Corresponding authors were contacted by email regarding missing follow-up data by E.Z. De novo head and neck carcinomas were defined as ear, nose, pharynx, larynx, lip, oral (gland), buccal, tongue, or tonsil carcinomas. Likewise, de novo colorectal carcinoma was defined as colon and rectal carcinomas. De novo uterine carcinoma included uterus and cervix carcinomas. The types of immunosuppressive drugs recorded for the included articles were AZA, CsA, MMF, TAC, SIR, and EVER.

### 2.3. Quality Assessment

To assess the quality of the included articles, the Newcastle–Ottawa Scale was consulted as risk of bias tool [12]. The coding manual for cohort studies was used by allocating stars for included articles to assess bias in selection, comparability of the study groups, and outcome of interest. The assessment was performed by A.P. and E.Y. independently. Conflicts were solved through discussion.

### 2.4. Statistical Analysis

The incidence of de novo carcinomas per 1000 person-years was calculated and pooled with DerSimonian–Laird random effects model in RevMan 5 [13]. The odds ratio (OR) for developing a de novo carcinoma between different immunosuppressive drugs was calculated with the Mantel–Haenszel random effects test in RevMan 5. Forest plots display the included studies for each comparison, with the OR per solid organ transplantation type and the overall effect presented with a 95% confidence interval. Events were defined as the occurrence of a de novo carcinoma. A *p* value less than or equal to 0.05 was considered statistically significant. All possible comparisons of immunosuppressive drugs present in the included cohorts were tested. Outcomes of comparisons with two or more study cohorts were considered eligible.

## 3. Results

### 3.1. Study Selection

**Figure 1:**
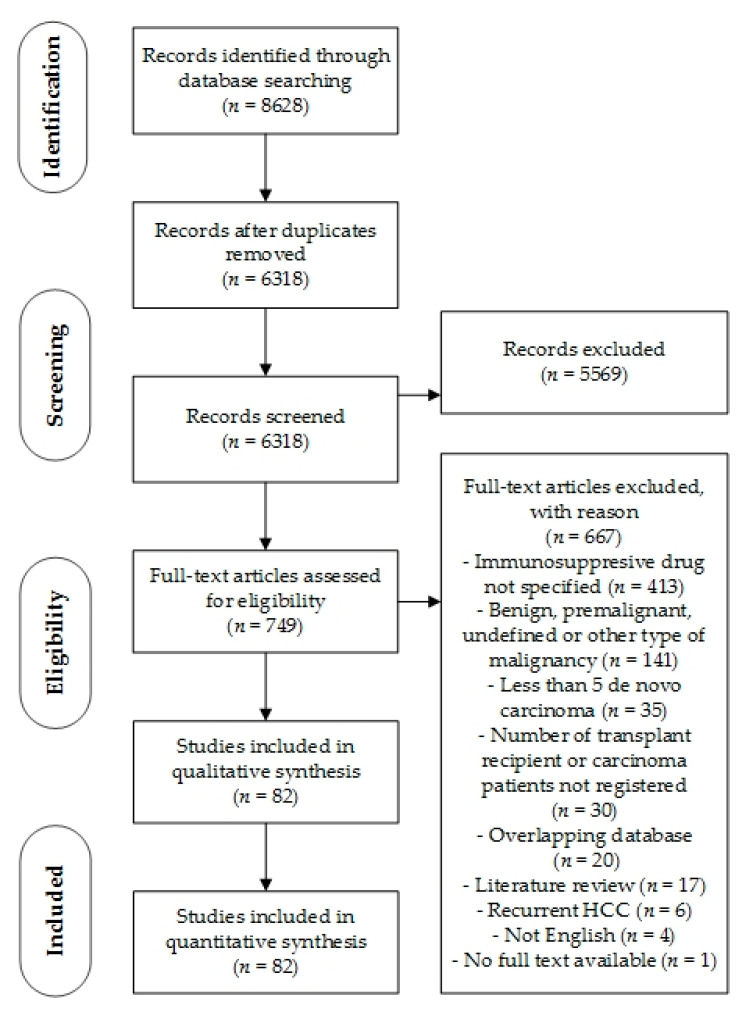
PRISMA Flowchart.

After duplicate removal, the search identified 6318 records. Based on title and abstract, 5569 records were excluded. Consequently, 749 full-text articles were assessed for eligibility. After exclusion of 667 articles, a total of 82 were included for qualitative and quantitative synthesis (Figure 1).

### 3.2. Study Characteristics

Overall, these 82 studies comprised a total of 237,540 recipients, who received 207,304 kidney, 21,404 liver, 5865 heart, and 2235 lung transplants. Transplant recipients were followed up for a mean period of 84.8 months after transplantation. Patients were diagnosed with a de novo carcinoma at a mean age of 52.3 years and after 66.8 months of follow-up. The baseline characteristics of the included cohorts are presented in Table 2. Most of the 82 studies were conducted in the United States (*n* = 11), followed by France (*n* = 9), Italy (*n* = 7), and Korea (*n* = 6) (Table S2). The vast majority of the studies (*n* = 64) were hospital-based, while others were database-guided or multicenter studies. Thirty-two authors were contacted regarding missing follow-up data, of which only 3 replied and 10 had invalid contact information.

### 3.3. Quality Assessment

Overall, 33 of the 82 studies scored 5 or 6 out of a maximum of 8 points, which represents a fair quality. A score of 5 or 6 was mainly due to missing information regarding follow-up in the outcome category and the ascertainment of exposure in the selection category. The remaining 49 articles were considered high-quality studies with a score equal to or over 7 (Table S3).

### 3.4. De Novo Carcinoma Occurrence

**Figure 2:**
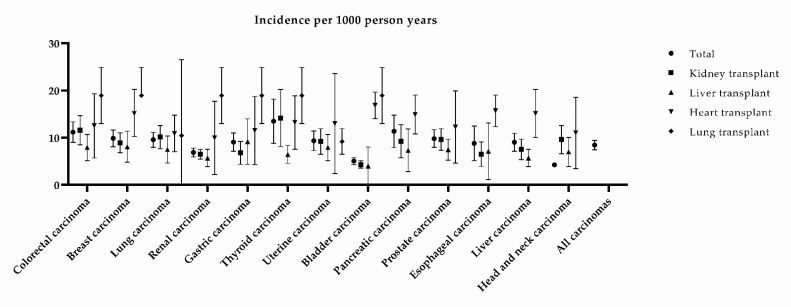
Pooled mean incidence of de novo carcinomas per 1000 person-years. Bars denote the 95% confidence interval.

The incidence rate per 1000 person-years of solid organ transplant recipients developing de novo carcinomas was 8.41 (95% CI 7.40–9.43, *p* < 0.00001). De novo carcinoma occurrence in the included studies varied from 7.81 to 115.4 cases per 1000 person-years. Patients who underwent a heart transplantation developed more de novo carcinomas compared to kidney and liver transplantations, particularly de novo bladder and upper gastrointestinal tract carcinomas (Figure 2).

### 3.5. CsA Versus TAC, AZA, MMF, and SIR

Patients who received CsA had a significantly higher likelihood of developing a de novo carcinoma compared to patients who received TAC, both calcineurin inhibitors (OR 1.56, 95% CI 1.00–2.44, *p* = 0.05) (Figure S1). No difference was found in the subgroup analysis for different types of de novo carcinomas. The odds for development of de novo carcinoma were not significantly different for patients who received CsA compared to AZA, one of the antimetabolites (OR 1.04, 95% CI: 0.90–1.21, *p* = 0.59), yet there appeared to be a trend towards higher occurrence of de novo esophageal and duodenal carcinoma in patients who received CsA (OR 2.47 95% CI 0.62–9.77, *p* = 0.20 and OR 4.05 95% CI 0.42–39.23, respectively) (Figure S2). Significantly more de novo carcinomas developed in patients who received CsA compared to patients who received MMF, another antimetabolite (OR 1.26, 95% CI 1.03–1.56, *p* = 0.03) (Figure S3). There was no difference observed in the subgroup analysis, but there appeared to be a trend of a higher likelihood of developing de novo head and neck carcinomas in patients who received CsA (OR 2.68, 95% CI 0.83–8.65, *p* = 0.10). There was no significant difference between patients using CsA and SIR, an mTOR inhibitor (OR 1.29, 95% CI 0.70–2.36, *p* = 0.87) (Figure S4).

### 3.6. AZA Versus TAC and MMF

Patients who received AZA had a higher likelihood of developing de novo head and neck carcinomas compared to patients who received TAC (OR 3.78, 95% CI 1.11–12.83, *p* = 0.03). Furthermore, there appeared to be a trend for a higher overall likelihood of developing a de novo carcinoma and for developing de novo lung carcinoma (OR 2.00, 95% CI 0.78–5.14, *p* = 0.15) (OR 7.28, 95% CI 0.93–56.73, *p* = 0.06) (Figure S5). Patients who received AZA had significantly higher odds of developing de novo carcinomas compared to patients who received MMF (OR 3.34, 95% CI 1.29–8.65, *p* = 0.01) (Figure S6).

### 3.7. MMF Versus TAC

No difference was observed in development of de novo carcinomas between patients who received MMF and patients who received TAC (OR 0.88, 95% CI 0.69–1.14, *p* = 0.33) (Figure S7).

## 4. Discussion

This systematic review shows that patients who receive immunosuppressive drugs after solid organ transplantation have a high incidence of de novo carcinomas, with an almost 20-fold increase compared to the age-corrected general population, as indicated by the WHO Global incidence of cancer between ages 30 and 69 (0.43 cases per 1000 person-years) [14]. This age range is comparable with the range described in the included studies. The incidence found in the analyzed cohorts for each specific type of de novo carcinoma resulted in a particularly high likelihood of de novo bladder and upper gastrointestinal tract carcinomas after heart transplantation. The incidence of bladder carcinomas in the general population between ages 30 and 69 is 0.075 per 1000 person-years, which is over 200 times lower than after a heart transplantation [14]. Heart transplantation recipients typically receive a higher dose of immunosuppressive drugs compared to kidney and liver transplantation recipients, causing a larger impairing effect on the immune system [7]. Furthermore, heart transplant recipients are described to be on average older and often tend to have a history of smoking, which are independent risk factors for bladder and upper gastrointestinal tract carcinomas [15,16,17]. However, the significant influence of the immunosuppressive therapy after heart transplantations cannot be ignored.

The current meta-analysis did not show a significant correlation between specific carcinomas and different immunosuppressive drugs, except for significantly less head and neck carcinomas in patients using the calcineurin inhibitor TAC or the antimetabolite MMF compared to the antimetabolite AZA. However, the comparison between MMF and AZA has to be interpreted carefully, as it was only described in two of the included studies, which consisted of unequal cohorts of patients. Many of the comparisons were rarely described in the included articles, which might cause those comparisons to be underpowered. Therefore, the lack of statistically significant results for those evaluations should not be considered irrelevant, but should warrant future epidemiological studies.

Furthermore, in agreement with previously published studies, the current results show that overall de novo carcinomas occur more often in solid organ transplant recipients using the calcineurin inhibitor CsA compared to MMF and TAC [18,19]. For instance, Tjon et al. showed that CsA treatment in comparison to TAC is the most important risk factor for de novo carcinoma in liver transplant recipients, supporting the results that this review provides for the whole transplant population [18]. Pathogenesis of specific types of cancer may be clarified by looking in depth into which immunosuppressive agents induce carcinogenesis.

Calcineurin inhibitors CsA and TAC are considered to have a similar working mechanism on the immune system via the calcineurin pathway. The main working mechanism is inhibition of the calcineurin activity in immune cells, thereby preventing the activation and nuclear translocation of nuclear factor of activated T-cells (NFAT), leading to inhibition of Interleukin-2 (IL-2) production in T cells [20]. IL-2 is an important factor for maintenance of CD4+ regulatory T cells, but also plays a critical role in the proliferation and differentiation of CD4+ T cells, promotion of CD8+ T cell and NK cell cytotoxic activity, and modulation of T cell differentiation programs in response to tumor antigens [21]. Inhibition of IL-2 production therefore has a profound effect on the immune system. In vitro and in vivo, calcineurin inhibitors inhibited degranulation of NK cells and reduced IFNy production by NK cells [22]. Furthermore, the capability of dendritic cells to stimulate T cells and produce IL-12 and CXC-chemokine ligand 10 is reduced [23,24]. If dendritic cells are incubated with tacrolimus, they develop a tolerogenic phenotype, which has a suppressive effect on CD4+ T cell proliferation [25].

Both CsA and TAC promote tumor formation by inducing tumor growth factor-β (TGF-β) and inhibiting apoptosis and DNA repair. This results in enhanced growth and diminished apoptosis of cancer cells [18,26,27,28]. The discrepancy between tumor-promoting effects of CsA and TAC might be due to the lower level of TGF-β that is induced by TAC compared to CsA [26,29]. In a healthy cell, TGF-β is a multifunctional cytokine that hampers proliferation, promotes apoptosis, and induces differentiation and fibroblast growth. However, in carcinoma cells, TGF-β loses its controlling function, leading to enhanced proliferation, diminished differentiation, and apoptosis of carcinoma cells [30].

Additionally, in vivo CsA has been shown to induce tumor progression and angiogenesis, independent of calcineurin, by releasing mitochondrial reactive oxygen species, leading to stimulation of mitogenic pathways in tumor and stromal cells [31]. Another mechanism via which CsA promotes angiogenesis is stimulation of prolyl hydroxylase activity, causing hypoxia-inducible factor 1a (HIF-1α) destabilization. HIF1α increases the expression of vascular endothelial growth factor, leading to angiogenesis [32].

Moreover, patients using CsA also have a higher incidence of Kaposi sarcoma and lymphoma [33,34]. Taking into account the effects of oncogenic viruses such as Epstein–Barr virus on non-Hodgkin lymphoma and human immunodeficiency virus on Kaposi sarcoma, this result insinuates a greater role of the suppressed immune system on the oncogenic effect of CsA than a direct effect of CsA on epithelial cells.

Both CsA and AZA give a higher odds to develop de novo carcinomas compared to MMF, even though MMF is more potent in preventing graft rejection than AZA [6,35]. Both AZA and MMF inhibit the purine pathway, but do so via different metabolites. MMF ultimately inhibits the formation of guanine nucleotides [36]. Guanine is one of the purine nucleobases necessary to generate DNA and is thus required for cell replication. MMF is metabolized to mycophenolic acid (MPA), which inhibits the enzyme inosine monophosphate dehydrogenase, thereby reducing the amount of guanine nucleotides formed [36]. Cells are able to generate guanine nucleotides through two distinct pathways: the de novo pathway and the salvage pathway. Whereas other cells are able to use both pathways, lymphocytes are only able to use the de novo pathway, and thus their proliferation is inhibited. Not only lymphocytes but also fibroblasts are affected by MMF, which are suspected to also rely partly on the de novo pathway. AZA also has an effect on the de novo purine synthesis pathway. AZA is a prodrug of 6-mercaptopurine (6-MP) and is nonenzymatically cleaved into 6-MP and imidazole derivatives. The main therapeutic effect of AZA relies on its metabolism to cytotoxic thioguanine nucleotides via the 6-MP pathway, which also inhibit the de novo purine synthesis, by inhibiting amidotransferase enzymes and purine ribonucleotide interconversion [37,38]. In addition, toxic thioguanine nucleotides are incorporated into DNA and RNA [37,38], which is thought to mediate the cytotoxic effects of AZA. Furthermore, the imidazole derivatives potentially also have effects on lymphocyte function. Although this metabolite has not been investigated in relation to the therapeutic effects of AZA, imidazole derivatives can reduce T cell proliferation and NFAT signaling following T cell receptor activation in mice [39]. Moreover, AZA can directly promote apoptosis and inhibit proliferation pathways through inhibition of Rac1 and Bcl-xL [40,41], and via inhibition of Rac1, it also blocks CD28 signaling [42]. In addition to its action on T cells, 6-MP can also inhibit Rac1 in activated macrophages, which leads to a reduction in the expression of inducible nitric oxide synthase [40]. The Rac1 pathway is also targeted by 6-MP in nonimmune cells [43], as 6-MP decreases Rac1 activation in endothelial cells and reduces activation of nuclear factor κ-light-chain-enhancer of activated B cells (NF-κB), leading to decreased transcription of proinflammatory cytokines. Furthermore, 6-MP selectively decreases VCAM-1 protein levels in TNF-α stimulated endothelial cells [44]. The elaborate effects of AZA, not only on the lymphocytes but also on macrophages and endothelial cells, might cause the higher odds of developing a de novo carcinoma compared to MMF.

The final group of drugs in this meta-analysis are the mTOR inhibitors. Aside from lymphocytes proliferation inhibition, mTOR inhibitors play a role in the intracellular signaling pathways in all cells of the immune system. For instance, the lifespan of and expression of costimulatory molecules by dendritic cells are increased, while on the other hand, metabolic NK cell function is reduced by mTOR inhibition [45].

As many malignancies upregulate the mTOR pathway, the mTOR inhibitors are currently used as anticancer therapeutics [46]. In this meta-analysis, there was no significant difference between mTOR inhibitors and other immunosuppressive drugs. However, there were only five studies which described the use of sirolimus in comparison with another drug and none which described everolimus. Therefore, perhaps there was insufficient power to detect any differences.

In the first months after transplantation, the risk of graft rejection is highest. Therefore, patients receive induction therapy in the first period after solid organ transplantation. In the included articles, the induction therapy regimen is scarcely described. Each center decides the optimal regimen based on the patient’s characteristics, but induction therapy mostly consists of a triple therapy combination of corticosteroids, IL-2 receptor antagonists, polyclonal antilymphocyte and antithymocyte preparations, and monoclonal antibody targeting. The effect of solely the induction therapy on carcinogenesis is still unknown. A Cochrane review from 2017 regarding the polyclonal and monoclonal antibody therapies showed an uncertain effect on malignancies [47]. It has also been described that basiliximab, an IL-2 receptor antagonist, does not increase the risk of malignancies [48]. The effect on carcinoma formation rather than malignancies in general has not been investigated separately. However, the increased odds of developing de novo carcinomas found in this meta-analysis are therefore most likely caused by the maintenance therapy.

Within the immunosuppressive regimen, patients often switch to different drugs in case of chronic rejection, adverse events, or the availability of new drugs. Four years after the transplantation, less than half of the patients still used the first prescribed combination [49]. However, this study also included the patients who received induction therapy, which explains why the majority of switches were found in the first year. In this meta-analysis, correction for switches in the maintenance therapy was not possible. Therefore, only studies describing longer periods of baseline therapy were included in the drug-specific comparisons. Furthermore, patients use combination triple therapies. For most of the patients, the baseline therapy was described in the included articles, and only for 689 patients the prescribed triple therapy combination. Even though only the baseline therapies were included in the drug-specific comparisons, one can assume that over time, multiple other drugs were simultaneously given. The effect of these switches on the outcome cannot be assessed, but as switches would have occurred in each group, these might partially cancel each other out.

There are many other factors contributing to the carcinogenesis process, such as smoking, alcohol, diet, and genetics. For certain carcinomas, including HCC and cervix carcinomas, viruses can also play a pivotal role in the carcinogenesis process. For HCC, hepatitis B virus (HBV) causes a 100-fold increase of the relative risk to develop HCC. The oncogenic role of HBV is not completely understood as it might be caused by both direct and indirect mechanisms, including immune-mediated hepatic inflammation leading to genetic damage, the induction of oxidative stress, and integration of the HBV DNA into the host genome that induces chromosomal instability [48]. Due to the immunosuppressive drugs, HBV can be reactivated. For cervix carcinoma, human papilloma virus HPV plays an important role. Furthermore, also vulva, vagina, penis, and anus carcinomas are associated with HPV [50]. In a systematic review by Grulich et al. [51], they reported increased standardized incidence ratios for these HPV-associated carcinomas in patients who underwent a solid organ transplantation. Unfortunately, the occurrence of viruses was not well described in the included articles. One might assume that this plays an additional role in the carcinogenesis which could not be corrected for in the meta-analysis.

Most studies had a long inclusion period, which might lead to differences in treatment regimens. The longest study had an inclusion period of 47 years and in another study, the first included patient received their solid organ transplantation in 1963. Since then, a lot has changed in the knowledge and possibilities of immunosuppressive drugs. A major breakthrough was the discovery of cyclosporine A, which was first given to patients in 1978 [52]. Furthermore, the detection of de novo carcinomas has improved with better imaging techniques and standardized follow-up protocols. In this study, there was no trend towards a higher or lower incidence per 1000 person-years of de novo carcinomas based on year of publication. In the comparisons of different immunosuppressive drugs, the longer inclusion period probably has a minimal influence as the inclusion period is equal for both drugs within one article.

This systematic review has several limitations. Many of the cohort studies included were conducted retrospectively, based on small groups of transplant recipients, and were often hospital-based. Small cohorts might lead to overestimation of the effect. Even though articles with less than five de novo carcinomas were excluded, this might still have introduced some overestimation. Using the NOS score, most articles were deemed to be of fair quality. One of the major problems was lacking information regarding follow-up data. Even though all authors were contacted regarding missing follow-up data, this might still introduce some risk of bias as not all authors replied to supply the follow-up data. These missing follow-up data also limit direct comparability of calculated incidences to the general population. However, the clear trend towards a higher incidence of carcinoma after solid organ transplantation cannot be ignored. Furthermore, large (inter-)national registries and studies based on International Classification of Diseases (ICD) codes might have missed a few de novo carcinomas as diagnosis might not always be coded correctly in the patient records. This could lead to underrepresentation of de novo carcinoma occurrence and bias in the outcome category. Additionally, changes in the immunosuppressive drug regimens could have been missed. A thorough check through each individual patient record is the only way to prevent missing changes in therapy leading to ascertainment bias and missing de novo carcinomas leading to assessment of exposure bias. Many studies were not eligible due to the strict requirements that the immunosuppressive drug regimen and the total solid organ transplantation group described. Finally, the changes in maintenance therapies were rarely described, while this might also influence the cancer development in the long term.

To determine the specific correlation between immunosuppressive drugs and cancer development, a combination of a large prospective cohort with sufficient follow-up for carcinomas to develop and translational research is needed. Important confounders can be determined from the prospective cohorts and further examined in in vitro and in vivo models.

Looking in depth into pathways of calcineurin inhibitors, such as IL-2, TGF-B, and HIF1α, and antimetabolites pathways may lead to enhanced comprehension of carcinogenesis in transplant recipients. Additionally, as described, these pathways are also contributing to carcinogenesis in the general population. Exploring these pathways would thus be an interesting topic for translational research and could in the long term give rise to preventive and therapeutic options for specific types of cancer, both in patients who underwent a solid organ transplantation and in the general population.

## 5. Conclusions

This systematic review and meta-analysis show an almost 20-fold higher likelihood of de novo carcinoma development in patients using immunosuppressive drugs after solid organ transplantation. The likelihood is highest for patients receiving cyclosporine A and azathioprine. By looking in depth into the pathways affected by these immunosuppressive drugs, a deeper understanding of carcinogenesis can be achieved and new starting points for translational and clinical research might be found.

## Figures and Tables

**Table 1:** Class of inhibitors and main working mechanisms.

Class of Inhibitor	Main Mechanism of Action	Immunosuppressive Drug
Calcineurin inhibitor	Inhibition of T cell proliferation	Cyclosporine A Tacrolimus
Antimetabolites	Inhibition DNA synthesis	Azathioprine Mofetil mycophenolate
mTOR inhibitors	Inhibition of mTOR kinase, involved in metabolism, growth, and proliferation	Sirolimus Everolimus

**Table 2:** Baseline characteristics of transplant recipients.

Variables	Transplant Recipients
Total solid organ transplant recipients, *n*	237,540
Kidney transplant	207,304
Liver transplant	21,404
Heart transplant	5865
Lung transplant	2235
Other transplant	732
Follow-up (in months), mean	84.8
	**Patients with PTC**
Sex (M/F), *n*	1782/698
Time until diagnosis (in months), mean	66.8 (73/82)
Age at diagnosis, mean	52.3 (42/82)
Living/cadaveric donor, *n*	545/1642 (28/82)
Smokers, *n*	250
Induction therapy, *n*	172
Baseline immunosuppressive therapy, *n*	
AZA	723
MMF	741
CsA	1055
TAC	627
SIR	201
EVER	6
Combined triple therapies, *n*	
CsA + AZA + steroids	296
CsA + MMF + steroids	90
TAC + AZA + steroids	10
TAC + MMF + steroids	203
CsA + SIR + steroids	90
Survival	
1-year (%)	81.3 (12/82)
3-year (%)	75.5 (6/82)
5-year (%)	62.4 (16/82)

Numbers are calculated for studies including these variables, (*n*/82) shows number of studies used for calculation. Abbreviations: PTC: post-transplant de novo carcinoma | M/F: male/female | MMF: Mycophenolate mofetil | CsA: Cyclosporine A | TAC: Tacrolimus | SIR: Sirolimus | EVER: Everolimus.

## Supplementary Materials

The following are available online at https://www.mdpi.com/2072-6694/13/5/1122/s1, Table S1: Search strategy, Table S2: Study characteristics, Table S3: Newcastle–Ottawa Scale risk of bias score, Figure S1: Cyclosporine A versus tacrolimus, Figure S2: Cyclosporine A versus azathioprine, Figure S3: Cyclosporine A versus mycophenolate mofetil, Figure S4: Cyclosporine A versus sirolimus, Figure S5: Azathioprine versus tacrolimus, Figure S6: Azathioprine versus mycophenolate mofetil, Figure S7: Mycophenolate mofetil versus tacrolimus.
