# Supplementary material for: De Novo Carcinoma after Solid Organ Transplantation to Give Insight into Carcinogenesis in General—A Systematic Review and Meta-Analysis
Source: Cancers (Basel). 2021 Mar 5;13(5):1122. doi: 10.3390/cancers13051122 (PMC7961956; doi:10.3390/cancers13051122)
Supplement: Supplementary file 1 [file cancers-13-01122-s001.pdf]

cancers

MDPI

Supplementary Materials: De Novo Carcinoma after Solid Organ Transplantation to Give Insight in Carcinogenesis in General, A Systematic Review and Meta-Analysis

Eline S. Zwart, Esen Yüksel, Anne Pannekoek, Ralph de Vries, Reina E. Mebius and Geert Kazemier

Table S1. Search strategy.

Pubmed September 10th 2020		
**Search**	**Query**	**Items found**
#8	Search (#7 NOT (animals[mh] NOT humans[mh]))	3516
#7	Search (#6 NOT (("Adolescent"[Mesh] OR "Child"[Mesh] OR "Infant"[Mesh] OR adolescen*[tiab] OR child*[tiab] OR schoolchild*[tiab] OR infant*[tiab] OR girl*[tiab] OR boy*[tiab] OR teen[tiab] OR teens[tiab] OR teenager*[tiab] OR youth*[tiab] OR pediatr*[tiab] OR paediatr*[tiab] OR puber*[tiab]) NOT ("Adult"[Mesh] OR adult*[tiab] OR man[tiab] OR men[tiab] OR woman[tiab] OR women[tiab])))	3546
#6	Search #4 and #5	3737
#5	Search ("Epidemiologic Studies"[Mesh] OR cohort[tiab] OR (case[tiab] AND (control[tiab] OR controll*[tiab] OR comparison[tiab] OR referent[tiab])) OR risk[tiab] OR causation[tiab] OR causal[tiab] OR "odds ratio"[tiab] OR etiol*[tiab] OR aetiol*[tiab] OR "natural history"[tiab] OR predict*[tiab] OR prognos*[tiab] OR outcome[tiab] OR course[tiab] OR retrospect*[tiab] OR followup[tiab] OR "follow up"[tiab])	7,007,005
#4	Search #1 AND #2 AND #3	5608
#3	Search "Carcinoma"[Mesh] OR "Sarcoma"[Mesh] OR "Melanoma"[Mesh] OR adenocarcinoma*[tiab] OR carcinoma*[tiab] OR sarcoma*[tiab] OR melanoma*[tiab] OR malign*[tiab]	1,628,750
#2	Search ("Organ Transplantation"[Mesh] OR transplant*[tiab] OR graft*[tiab] OR allotransplant*[tiab] OR allograft*[tiab] OR posttransplant*[tiab]) AND (heart[tiab] OR kidney[tiab] OR renal[tiab] OR liver[tiab] OR lung[tiab] OR solid organ*[tiab])	280,522
#1	Search "Immunosuppression"[Mesh] OR "Immunosuppressive Agents"[Mesh] OR immunosuppress*[tiab] OR immuno-suppress*[tiab] OR immunesuppress*[tiab] OR immune-suppress*[tiab] OR immunodepress*[tiab] OR immunedepress*[tiab] OR immuno-depress*[tiab] OR immune-depress*[tiab] OR allotype suppress*[tiab] OR antibody induction[tiab]	260,506

Embase September 10th 2020		
**Search**	**Query**	**Items found**
#9	#8 NOT (('adolescent'/exp OR 'child'/exp OR adolescent*:ti,ab OR child*:ti,ab OR schoolchild*:ti,ab OR infant*:ti,ab OR girl*:ti,ab OR boy*:ti,ab OR teen:ti,ab OR teens:ti,ab OR teenager*:ti,ab OR youth*:ti,ab OR pediatr*:ti,ab OR paediatr*:ti,ab OR puber*:ti,ab) NOT ('adult'/exp OR 'aged'/exp OR 'middle aged'/exp OR adult*:ti,ab OR man:ti,ab OR men:ti,ab OR woman:ti,ab OR women:ti,ab))	5561
#8	#6 AND #7	5915
#7	'epidemiology'/de OR cohort:ti,ab,kw OR (case:ti,ab,kw AND (control:ti,ab,kw OR controll*:ti,ab,kw OR comparison:ti,ab,kw OR referent:ti,ab,kw)) OR risk:ti,ab,kw OR causation:ti,ab,kw OR causal:ti,ab,kw OR 'odds ratio':ti,ab,kw OR etiol*:ti,ab,kw OR aetiol*:ti,ab,kw OR 'natural history':ti,ab,kw OR predict*:ti,ab,kw OR prognos*:ti,ab,kw OR outcome:ti,ab,kw OR course:ti,ab,kw OR retrospect*:ti,ab,kw OR followup:ti,ab,kw OR 'follow up':ti,ab,kw	8,819,132
#6	#5 NOT ([animals]/lim NOT [humans]/lim)	9477
#5	#4 NOT (conference*:it OR letter*:it OR editorial*:it)	9804
#4	#1 AND #2 AND #3	15,194
#3	'carcinoma'/exp OR 'sarcoma'/exp OR 'melanoma'/exp OR adenocarcinoma*:ab,ti,kw OR carcinoma*:ab,ti,kw OR sarcoma*:ab,ti,kw OR melanoma*:ab,ti,kw OR malign*:ab,ti,kw	2,365,914
#2	'solid organ transplantation'/exp OR 'solid organ transplant'/exp OR 'solid organ transplant recipient'/exp OR (('organ transplantation'/exp OR transplant*:ab,ti,kw OR graft*:ab,ti,kw OR allotransplant*:ab,ti,kw OR allograft*:ab,ti,kw OR posttransplant*:ab,ti,kw) AND (heart:ab,ti,kw OR kidney:ab,ti,kw OR renal:ab,ti,kw OR liver:ab,ti,kw OR lung:ab,ti,kw OR 'solid organ*':ab,ti,kw))	455,808

#1	'immunosuppressive treatment'/exp OR 'immunosuppressive agent'/exp OR immunosuppress*:ab,ti,kw OR 'immuno-suppress*':ab,ti,kw OR immunesuppress*:ab,ti,kw OR 'immune-suppress*':ab,ti,kw OR immunodepress*:ab,ti,kw OR immunedepress*:ab,ti,kw OR 'immuno-depress*':ab,ti,kw OR 'immune-depress*':ab,ti,kw OR 'allotype suppress*':ab,ti,kw OR 'antibody induction':ab,ti,kw	1,246,113

Table S2. Study characteristics * Follow up after transplantation (months): mean unless marked with † (median). ** Only reported for the patients with post-transplant malignancy. Abbreviations: NR: Not reported ∣ USRDS: United States Renal Data System ∣ OPTN: Organ Procurement and Transplantation Network ∣ UNOS: United Network for Organ Sharing ∣ ANZLTR: Australian & New Zealand Liver Transplant Registry ∣ ANZCOTR: Australian and New Zealand Cardiothoracic Organ Transplant Registry ∣ CST: Canadian Society of Transplantation ∣ ANZDATA: Australian and New Zealand Dialysis and Transplant registry ∣ SRTR: Scientific Registry of Transplant Recipients.

Author	Country	Cohort	Total transplantations	Type of transplantation	Followup (months)*	Patients with de novo carcinoma, n (%)**	Type of carcinoma	Male (%)**
Aigner et al.[1]	Austria (1986–2005)	Hospital-based	3595	Solid organ	88	206 (5,73)	Any	76,9
**Albright et al.[2]**	US (1998–2001)	Hospital-based	402	Liver	62,4†	77 (19,15)	Any	NR
**Arichi et al.[3]**	Japan (1973–2007)	Hospital-based	429	Kidney	202	57 (13,29)	Any	63,2
**Ativitavas et al.[4]**	Thailand (1992–2007)	Hospital-based	270	Kidney	NR	18 (6,67)	Any	61,1
**Baccarani et al.[5]**	Italy (1991–2005)	Multicentre	417	Liver	81,6†	43 (10,31)	Any	74,4
**Bruschi et al.[6]**	Italy (1995–2011)	Hospital-based	878	Heart	135,6	22 (2,51)	Lung	90,9
**Campistol et al.[7]**	Multiple countries (1986–2005)	Multicentre	430	Kidney	60	17 (3,95)	Any	NR
**Carenco et al. [8]**	France (1991–2008)	Hospital-based	465	Liver	96	43 (9,25)	Any	NR
**Chambade et al.[9]**	France (2002–2006)	Hospital-based	2050	Kidney	17,4	7 (0,34)	Kidney	NR
**Chang et al.[10]**	Taiwan (2000–2015)	Hospital-based	222	Kidney	104	29 (13,1)	Bladder	NR
**Chen et al.[11]**	Taiwan (1992–2008)	Hospital-based	102	Kidney	45,5	9 (8,82)	Any	NR
**Chen et al.[12]**	Taiwan (2000–2012)	Multicentre	460	Kidney	75	31 (6,74)	Any	NR
**Couetil et al.[13]**	UK (1979–1988)	Hospital-based	303	Heart	NR	11 (3,63)	Any	100
**Cox et al.[14]**	US (1990–2010)	Hospital-based	5920	Kidney	45	11 (0,19)	Bladder	81,8
**Curtil et al.[15]**	France (1987–1996)	Hospital-based	267	Heart	NR	18 (6,74)	Any	94,4
**Davis et al.[16]**	Ireland (1976–2013)	Hospital-based	3741	Kidney	94	14 (0,37)	Bladder	NR
**Demir et al.[17]**	Turkey (2004–2009)	Hospital-based	100	Kidney	60	14 (14,00)	Any	NR
**Doublet et al.[18]**	France (1993–1995)	Hospital-based	129	Kidney	NR	5 (3,88)	Kidney	40
**Eccher et al.[19]**	Italy (1968–2015)	Hospital-based	1646	Kidney	100,8	16 (0,97)	Kidney	NR

**Egeli et al.[20]**	Turkey (1998–2016)	Hospital-based	429	Liver	103,4	9 (2,10)	Any	100
**Elkentaoui et al.[21]**	France (1983–2008)	Hospital-based	1350	Kidney	NR	36 (2,67)	Bladder	NR
**Filocamo et al.[22]**	Italy (1991–2007)	Hospital-based	694	Kidney	41	10 (1,44)	Kidney	70
**Fox et al.[23]**	Israel (1998–2012)	Hospital-based	412	Lung	60	39 (9,47)	Any	64,1
**Frezza et al.[24]**	US (1982–1992)	Hospital-based	1657	Liver	NR	50 (3,02)	Any	74
**Gallagher et al.[25]**	Australia (1983–1986)	Multicentre	481	Kidney	247,2†	226 (46,99)	Any	NR
**Heinz-Peer et al.[26]**	Austria (1993–1994)	NR	385	Kidney	36	6 (1,56)	Kidney	100
**Hurst et al.[27]**	US (2000–2005)	USRDS	40,281	Kidney	36	368 (0,91)	Kidney	66,3
**Jonas et al.[28]**	Germany (1988–1994)	Hospital-based	458	Liver	50,0†	33 (7,21)	Any	48,5
**Kahan et al.[29]**	US (1993–2002)	Hospital-based	1008	Kidney	45,4	35 (3,47)	Any	85,7
**Kamal et al.[30]**	Egypt (1976–2006)	Hospital-based	1865	Kidney	78	7 (0,38)	Bladder	100
**Kanaan et al.[31]**	Belgium (1976–2011)	Hospital-based	2944	Kidney	60	14 (0,48)	Liver	85,7
**Kar-czewski et al.[32]**	Poland (1994–2011)	Hospital-based	836	Kidney	120	63 (7,54)	Any	NR
**Kauffman et al.[33]**	US (1996–2001)	OPTN/UNOS	33,249	Kidney	32,1	569 (1,71)	Any	71,4
**Kehinde et al.[34]**	UK (1975–1991)	Hospital-based	492	Kidney	72	27 (5,49)	Any	66,7
**Kellerman et al.[35]**	US (1994–2007)	Hospital-based	851	Heart	64	73 (8,58)	Any	83,6
**Keown et al.[36]**	Multiple countries (1992–1994)	Multicentre	497	Kidney	12	44 (8,85)	Any	NR
**Kim et al.[37]**	Korea (1975–1999)	Multicentre	10,029	Kidney	156,2	193 (1,92)	Any	56
**Kim et al.[38]**	US (1995–2005)	Hospital-based	539	Kidney	120	18 (3,34)	Any	NR
**Kwak et al.[39]**	Korea (1999–2011)	Hospital-based	2139	Kidney	144	142 (6,64)	Any	NR
**Lebkow-ska et al.[40]**	Poland (NR)	Hospital-based	44	Kidney	NR	5 (11,36)	Thyroid	NR
**Li et al.[41]**	China (1998–2006)	Multicentre	1612	Kidney	57	9 (0,56)	Bladder	22,2
**Lichten-berg et al.[42]**	Israel (2001–2014)	Hospital-based	718	Kidney	42,8	32 (4,46)	Any	68,8
**Liu et al.[43]**	China (2002–2009)	Hospital-based	722	Kidney	NR	14 (1,94)	Any	78,6

**Lopez-Pintor et al.[44]**	Spain (1989–2007)	Hospital-based	500	Kidney	59,7	6 (1,20)	Lip	100
**Marcen et al.[45]**	Spain (1979–2001)	Hospital-based	793	Kidney	75,4	95 (11,98)	Any	NR
**Mathew et al.[46]**	Multiple countries (NR)	Multicentre	1675	Kidney	24	44 (2,63)	Any	NR
**McGeown et al.[47]**	Ireland (1968–1998)	Hospital-based	868	Kidney	122,4	86 (9,91)	Any	NR
**Mizuno et al.[48]**	Japan (2002–2017)	Hospital-based	97	Liver	82,8	11 (11,34)	Any	90,9
**Na et al.[49]**	Australia (1984–2006)	ANZLTR, ANZCOTR	4141	Solid organ	63,0†	58 (1,40)	Lip	77,6
**Neuzillet et al.[50]**	France (1987–2003)	Hospital-based	933	Kidney	39	11 (1,18)	Kidney	90,9
**Nure et al.[51]**	Italy (2002–2012)	Hospital-based	225	Liver	NR	11 (4,89)	Any	NR
**Oezcelik et al.[52]**	Germany (2000–2009)	Hospital-based	761	Liver	70,3	5 (0,66)	Esophagus	80
**Opelz et al.[53]**	Multiple countries (1999–2013)	CST	4279	Kidney	50,4	374 (8,74)	Any	NR
**Palazzetti et al.[54]**	Italy (1988–2014)	Multicentre	3111	Kidney	NR	28 (0,90)	Bladder	67,9
**Park et al.(1)[55]**	Korea (1998–2008)	Hospital-based	1952	Liver	42	44 (2,25)	Any	79,5
**Park et al.(2)[56]**	Korea (2004–2007)	Hospital-based	509	Kidney	137	15 (2,95)	Stomach	66,7
**Park et al.[57]**	Korea (1991–2016)	Hospital-based	9776	Solid organ	240	13 (0,13)	Head and neck	92,3
**Ploussard et al.[58]**	France (1984–2006)	Hospital-based	2396	Kidney	43	12 (0,50)	Kidney	41,7
**Raeisi et al.[59]**	Iran (1991–2012)	Hospital-based	1487	Kidney	NR	64 (4,30)	Any	NR
**Rinaldi et al.[60]**	Italy (1985–1998)	Hospital-based	474	Heart	71,1	55 (11,60)	Any	89,1
**Saigal et al.[61]**	UK (1988–1999)	Hospital-based	1140	Liver	70,9	29 (2,54)	Any	72,4
**Schmidt et al.[62]**	Germany (1968–1994)	Hospital-based	868	Kidney	41,8	12 (1,38)	Any	50
**Shoji et al.[63]**	Japan (1997–2002)	Hospital-based	554	Liver	56	5 (0,90)	Lung	80
**Singh et al.[64]**	India (NR)	NR	1500	Kidney	NR	31 (2,07)	Head and neck	NR
**Slavis et al.[65]**	US (1963–1969)	Hospital-based	14	Kidney	22,5	7 (50,00)	Any	NR
**Snanoudj et al.[66]**	France (1983–2004)	Hospital-based	1267	Kidney	149,0†	150 (11,84)	Any	NR
**Sobieszczanska et al.[67]**	Poland (1987–2011)	Hospital-based	324	Heart	276	29 (8,95)	Any	82,8

**Stauch et al.[68]**	Germany (1986 - 1991)	Hospital-based	160	Heart	60	11 (6,88)	Any	100
**Sun et al.[69]**	Korea (1991–2010)	Hospital-based	1425	Kidney	194,4	5 (0,35)	Kidney	60
**Tillou et al.[70]**	France (1988–2009)	Multicentre	41,806	Kidney	38,1	79 (0,19)	Kidney	68,4
**Tiwari et al.[71]**	India (2006–2017)	Hospital-based	2100	Liver	NR	21 (1,00)	Any	95
**To-maszewski et al.[72]**	UK (1992–2007)	Hospital-based	5686	Solid organ	40	11 (0,19)	Bladder	100
**van Leeu-wen et al.[73]**	US (1982–2003)	ANZDATA	8162	Kidney	NR	203 (2,49)	Lip	84,4
**Vegso et al.[74]**	Hungary (1973–2012)	Hospital-based	3530	Kidney	159,3	232 (6,57)	Any	55,6
**Vogt et al.[75]**	Germany (1981–1986)	Hospital-based	598	Kidney	41,8	18 (3,01)	Any	NR
**Wang et al.[76]**	Taiwan (1990–2015)	Hospital-based	454	Heart	69,2	27 (5,95)	Any	96,3
**Wu et al.[77]**	Taiwan (1983–2003)	Hospital-based	730	Kidney	72,2	63 (8,63)	Any	NR
**Yanik et al.[78]**	US (1995–2009)	SRTR, OPTN	5687	Kidney	25,4	85 (1,49)	Any	NR
**Yilmaz et al.[79]**	Turkey (2000–2015)	Hospital-based	867	Kidney	112,9	59 (6,81)	Any	69,5
**Yoshimura et al.[80]**	Japan (1970–1979)	Hospital-based	110	Kidney	300	7 (6,36)	Any	NR
**Yserbyt et al.[81]**	Belgium (2010–2012)	Hospital-based	494	Lung	126	13 (2,63)	Lung	69,2
**Yu et al.[82]**	China (2005–2011)	Hospital-based	569	Liver	44,9	16 (2,81)	Any	81,3

Table S3. Newcastle-Ottowa score.

Author	Year	Selection	Comparability	Outcome	NOS score
Aigner et al.[1]	2007	☆☆☆☆	☆	☆☆	7
Albright et al.[2]	2010	☆☆☆☆	☆	☆☆☆	8
Arichi et al.[3]	2008	☆☆☆	☆	☆☆	6
Ativitavas et al.[4]	2008	☆☆☆☆	☆	☆☆	7
Baccarani et al.[5]	2010	☆☆☆	☆	☆☆	6
Bruschi et al.[6]	2013	☆☆	☆	☆☆	5
Campistol et al.[7]	2006	☆☆☆☆	☆	☆☆	7
Carenco et al. [8]	2015	☆☆☆	☆	☆☆☆	7
Chambade et al.[9]	2008	☆☆	☆	☆☆☆	6
Chang et al.[10]	2019	☆☆☆☆	☆	☆☆☆	8
Chen et al.[11]	2009	☆☆☆	☆	☆☆	6
Chen et al.[12]	2015	☆☆☆☆	☆	☆☆☆	8
Couetil et al.[13]	1990	☆☆☆☆	☆	☆☆	7
Cox et al.[14]	2011	☆☆☆	☆	☆☆	6
Curtil et al.[15]	1997	☆☆☆	☆	☆☆☆	7
Davis et al.[16]	2013	☆☆☆	☆	☆☆☆	7
Demir et al.[17]	2015	☆☆☆☆	☆	☆☆☆	8
Doublet et al.[18]	1997	☆☆☆☆	☆	☆☆	7
Eccher et al.[19]	2016	☆☆☆☆	☆	☆☆☆	8
Egeli et al.[20]	2017	☆☆☆☆	☆	☆☆	7
Elkentaoui et al.[21]	2010	☆☆☆	☆	☆☆	6
Filocamo et al.[22]	2009	☆☆☆	☆	☆☆	6
Fox et al.[23]	2017	☆☆☆	☆	☆☆	6
Frezza et al.[24]	1997	☆☆☆☆	☆	☆	6
Gallagher et al.[25]	2010	☆☆☆	☆	☆☆☆	7
Heinz-Peer et al.[26]	1995	☆☆☆☆	☆	☆☆	7
Hurst et al.[27]	2010	☆☆☆	☆	☆☆	6
Jonas et al.[28]	1997	☆☆☆☆	☆	☆☆	7
Kahan et al.[29]	2005	☆☆☆	☆	☆☆	6
Kamal et al.[30]	2008	☆☆☆	☆	☆☆☆	7
Kanaan et al.[31]	2017	☆☆	☆	☆☆☆	6
Karczewski et al.[32]	2012	☆☆☆☆	☆	☆☆☆	8
Kauffman et al.[33]	2005	☆☆☆	☆	☆☆	6
Kehinde et al.[34]	1994	☆☆☆☆	☆	☆☆	7
Kellerman et al.[35]	2009	☆☆☆☆	☆	☆☆☆	8
Keown et al.[36]	1996	☆☆☆	☆	☆☆	6
Kim et al.[37]	2010	☆☆☆	☆	☆☆☆	7
Kim et al.[38]	2002	☆☆☆☆	☆	☆☆☆	8
Kwak et al.[39]	2013	☆☆☆☆	☆	☆☆☆	8
Lebkowska et al.[40]	2003	☆☆☆	☆	☆☆☆	7
Li et al.[41]	2009	☆☆☆	☆	☆☆☆	7
Lichtenberg et al.[42]	2017	☆☆☆	☆	☆	5
Liu et al.[43]	2015	☆☆☆☆	☆	☆☆	7
Lopez-Pintor et al.[44]	2011	☆☆☆☆	☆	☆☆	7
Marcen et al.[45]	2003	☆☆☆	☆	☆☆	6
Mathew et al.[46]	2004	☆☆☆	☆	☆	5
McGeown et al.[47]	2000	☆☆☆	☆	☆☆☆	7

Mizuno et al.[48]	2018	☆☆☆	☆	☆☆	6
Na et al.[49]	2016	☆☆☆	☆	☆☆	6
Neuzillet et al.[50]	2005	☆☆☆☆	☆	☆☆	7
Nure et al.[51]	2013	☆☆☆☆	☆	☆☆	7
Oezcelik et al.[52]	2011	☆☆☆☆	☆	☆☆	7
Opelz et al.[53]	2016	☆☆☆☆	☆	☆☆	7
Palazzetti et al.[54]	2018	☆☆☆☆	☆	☆	6
Park et al.(1)[55]	2012	☆☆☆	☆	☆	5
Park et al.(2)[56]	2012	☆☆☆☆	☆	☆☆	7
Park et al.[57]	2018	☆☆☆☆	☆	☆☆	7
Ploussard et al.[58]	2012	☆☆☆	☆	☆☆	6
Raeisi et al.[59]	2013	☆☆☆☆	☆	☆☆	7
Rinaldi et al.[60]	2001	☆☆☆☆	☆	☆☆☆	8
Saigal et al.[61]	2002	☆☆☆☆	☆	☆☆	7
Schmidt et al.[62]	1995	☆☆☆☆	☆	☆	6
Shoji et al.[63]	2017	☆☆	☆	☆☆	5
Singh et al.[64]	2006	☆☆☆	☆	☆☆	6
Slavis et al.[65]	1990	☆☆☆☆	☆	☆☆	7
Snanoudj et al.[66]	2004	☆☆☆☆	☆	☆☆	7
Sobieszczanska et al.[67]	2013	☆☆☆☆	☆	☆☆☆	8
Stauch et al.[68]	1993	☆☆☆☆	☆	☆☆	7
Sun et al.[69]	2013	☆☆	☆	☆☆☆	6
Tillou et al.[70]	2012	☆☆☆	☆	☆☆	6
Tiwari et al.[71]	2020	☆☆☆☆	☆	☆☆	7
Tomaszewski et al.[72]	2011	☆☆	☆	☆☆	5
van Leeuwen et al.[73]	2009	☆☆☆☆	☆	☆☆	7
Vegso et al.[74]	2011	☆☆☆☆	☆	☆☆	7
Vogt et al.[75]	1990	☆☆☆☆	☆	☆	6
Wang et al.[76]	2016	☆☆☆	☆	☆☆	6
Wu et al.[77]	2004	☆☆☆☆	☆	☆☆☆	8
Yanik et al.[78]	2015	☆☆☆	☆	☆	5
Yilmaz et al.[79]	2016	☆☆☆☆	☆	☆☆☆	8
Yoshimura et al.[80]	2005	☆☆☆	☆	☆☆☆	7
Yserbyt et al.[81]	2012	☆☆☆☆	☆	☆☆	7
Yu et al.[82]	2014	☆☆☆	☆	☆	5

Study or Subgroup	Cyclosporin A Events	Cyclosporin A Total	Tacrolimus Events	Tacrolimus Total	Weight	Odds Ratio M-H, Random, 95% CI
**1.1.1 Colorectal carcinoma**						
Arichi et al. 2008	2	208	0	92	2.0%	2.24 [0.11, 47.12]
Demir et al. 2015	1	19	1	65	2.3%	3.56 [0.21, 59.69]
Marcen et al. 2003	2	510	0	159	2.0%	1.57 [0.07, 32.84]
**Subtotal (95% CI)**		**737**		**316**	**6.2%**	**2.37 [0.43, 13.13]**
Total events	5		1			
Heterogeneity: Tau² = 0.00; Chi² = 0.15, df = 2 (P = 0.93); I² = 0%						
Test for overall effect: Z = 0.99 (P = 0.32)						
**1.1.2 Breast carcinoma**						
Arichi et al. 2008	2	208	0	92	2.0%	2.24 [0.11, 47.12]
Demir et al. 2015	0	19	1	65	1.8%	1.10 [0.04, 28.16]
Jonas et al. 1997	1	300	2	103	3.0%	0.17 [0.02, 1.88]
**Subtotal (95% CI)**		**527**		**260**	**6.8%**	**0.57 [0.11, 2.93]**
Total events	3		3			
Heterogeneity: Tau² = 0.00; Chi² = 1.93, df = 2 (P = 0.38); I² = 0%						
Test for overall effect: Z = 0.67 (P = 0.50)						
**1.1.3 Lung carcinoma**						
Carenco et al. 2015	2	86	11	266	6.3%	0.55 [0.12, 2.54]
Herrero et al. 2013	1	8	6	42	3.4%	0.86 [0.09, 8.27]
Marcen et al. 2003	1	510	0	159	1.8%	0.94 [0.04, 23.17]
**Subtotal (95% CI)**		**604**		**467**	**11.5%**	**0.67 [0.21, 2.17]**
Total events	4		17			
Heterogeneity: Tau² = 0.00; Chi² = 0.15, df = 2 (P = 0.93); I² = 0%						
Test for overall effect: Z = 0.67 (P = 0.50)						
**1.1.4 Renal carcinoma**						
Arichi et al. 2008	3	208	3	92	5.8%	0.43 [0.09, 2.19]
Demir et al. 2015	1	19	1	65	2.3%	3.56 [0.21, 59.69]
Hurst et al. 2010	275	9834	225	25315	23.5%	3.21 [2.69, 3.83]
Marcen et al. 2003	3	510	0	159	2.1%	2.20 [0.11, 42.82]
**Subtotal (95% CI)**		**10571**		**25631**	**33.6%**	**1.89 [0.62, 5.75]**
Total events	282		229			
Heterogeneity: Tau² = 0.61; Chi² = 5.85, df = 3 (P = 0.12); I² = 49%						
Test for overall effect: Z = 1.13 (P = 0.26)						
**1.1.5 Gastric carcinoma**						
Arichi et al. 2008	1	208	1	92	2.3%	0.44 [0.03, 7.11]
Marcen et al. 2003	1	510	0	159	1.8%	0.94 [0.04, 23.17]
**Subtotal (95% CI)**		**718**		**251**	**4.1%**	**0.61 [0.07, 4.98]**
Total events	2		1			
Heterogeneity: Tau² = 0.00; Chi² = 0.12, df = 1 (P = 0.73); I² = 0%						
Test for overall effect: Z = 0.46 (P = 0.64)						
**1.1.6 Uterine carcinoma**						
Arichi et al. 2008	4	208	1	92	3.5%	1.78 [0.20, 16.19]
Marcen et al. 2003	3	510	0	159	2.1%	2.20 [0.11, 42.82]
**Subtotal (95% CI)**		**718**		**251**	**5.6%**	**1.92 [0.33, 11.29]**
Total events	7		1			
Heterogeneity: Tau² = 0.00; Chi² = 0.01, df = 1 (P = 0.91); I² = 0%						
Test for overall effect: Z = 0.72 (P = 0.47)						
**1.1.7 Prostate carcinoma**						
Arichi et al. 2008	1	208	0	92	1.8%	1.34 [0.05, 33.14]
Marcen et al. 2003	4	510	0	159	2.1%	2.83 [0.15, 52.93]
**Subtotal (95% CI)**		**718**		**251**	**3.9%**	**2.02 [0.23, 17.53]**
Total events	5		0			
Heterogeneity: Tau² = 0.00; Chi² = 0.12, df = 1 (P = 0.73); I² = 0%						
Test for overall effect: Z = 0.64 (P = 0.53)						
**1.1.8 Bladder carcinoma**						
Arichi et al. 2008	1	208	1	92	2.3%	0.44 [0.03, 7.11]
Demir et al. 2015	0	19	1	65	1.8%	1.10 [0.04, 28.16]
**Subtotal (95% CI)**		**227**		**157**	**4.1%**	**0.65 [0.08, 5.36]**
Total events	1		2			
Heterogeneity: Tau² = 0.00; Chi² = 0.18, df = 1 (P = 0.67); I² = 0%						
Test for overall effect: Z = 0.40 (P = 0.69)						
**1.1.9 Head and neck carcinoma**						
Arichi et al. 2008	2	208	0	92	2.0%	2.24 [0.11, 47.12]
Carenco et al. 2015	4	86	10	266	9.0%	1.25 [0.38, 4.09]
Marcen et al. 2003	0	510	1	159	1.8%	0.10 [0.00, 2.55]
Na et al. 2016	49	2784	1	847	4.2%	15.16 [2.09, 109.93]
van Leeuwen et al. 2009	97	35822	2	1802	7.2%	2.44 [0.60, 9.92]
**Subtotal (95% CI)**		**39410**		**3166**	**24.1%**	**2.00 [0.55, 7.34]**
Total events	152		14			
Heterogeneity: Tau² = 1.14; Chi² = 9.14, df = 4 (P = 0.06); I² = 56%						
Test for overall effect: Z = 1.05 (P = 0.30)						
**Total (95% CI)**		**54230**		**30750**	**100.0%**	**1.56 [1.00, 2.44]**
Total events	461		268			
Heterogeneity: Tau² = 0.21; Chi² = 32.19, df = 25 (P = 0.15); I² = 22%						
Test for overall effect: Z = 1.95 (P = 0.05)						
Test for subgroup differences: Chi² = 4.78, df = 8 (P = 0.78), I² = 0%						

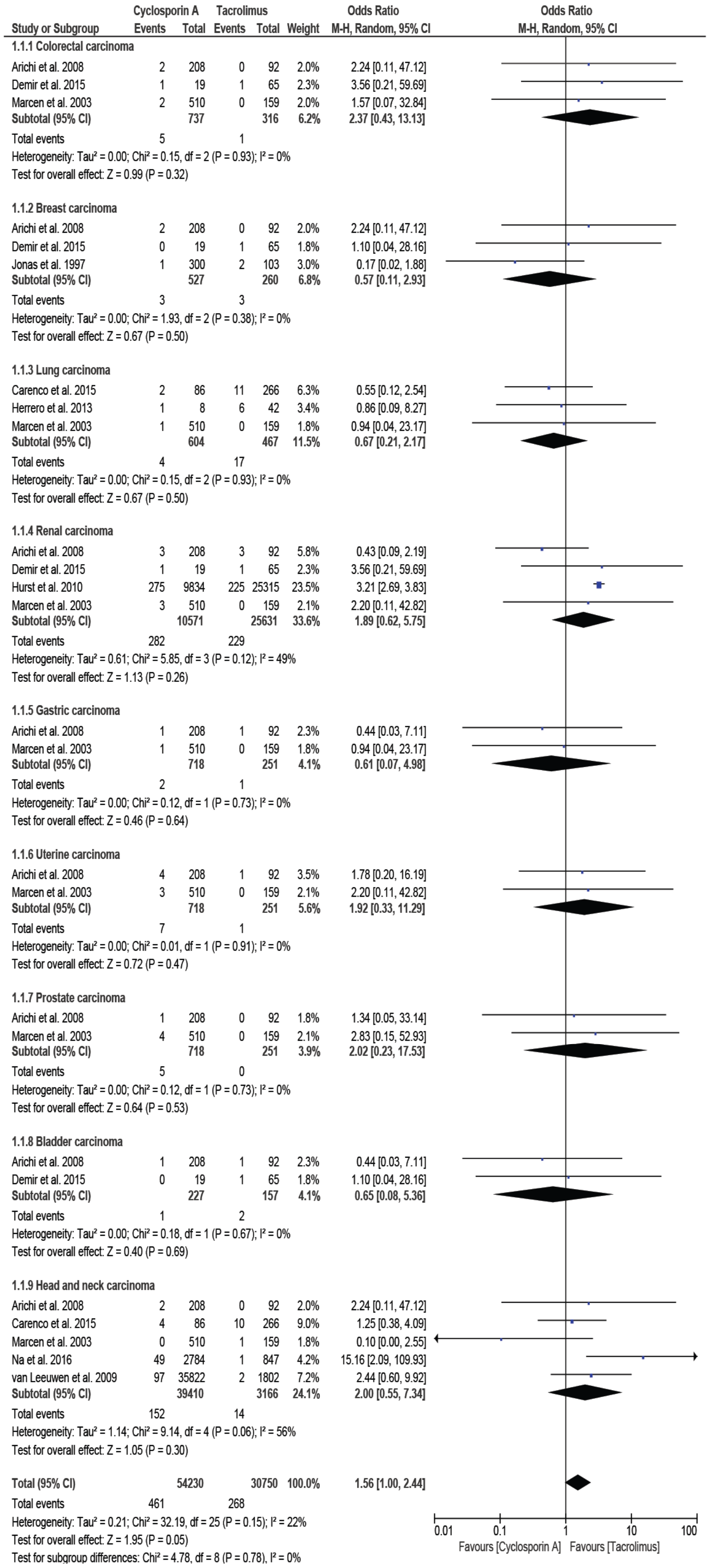

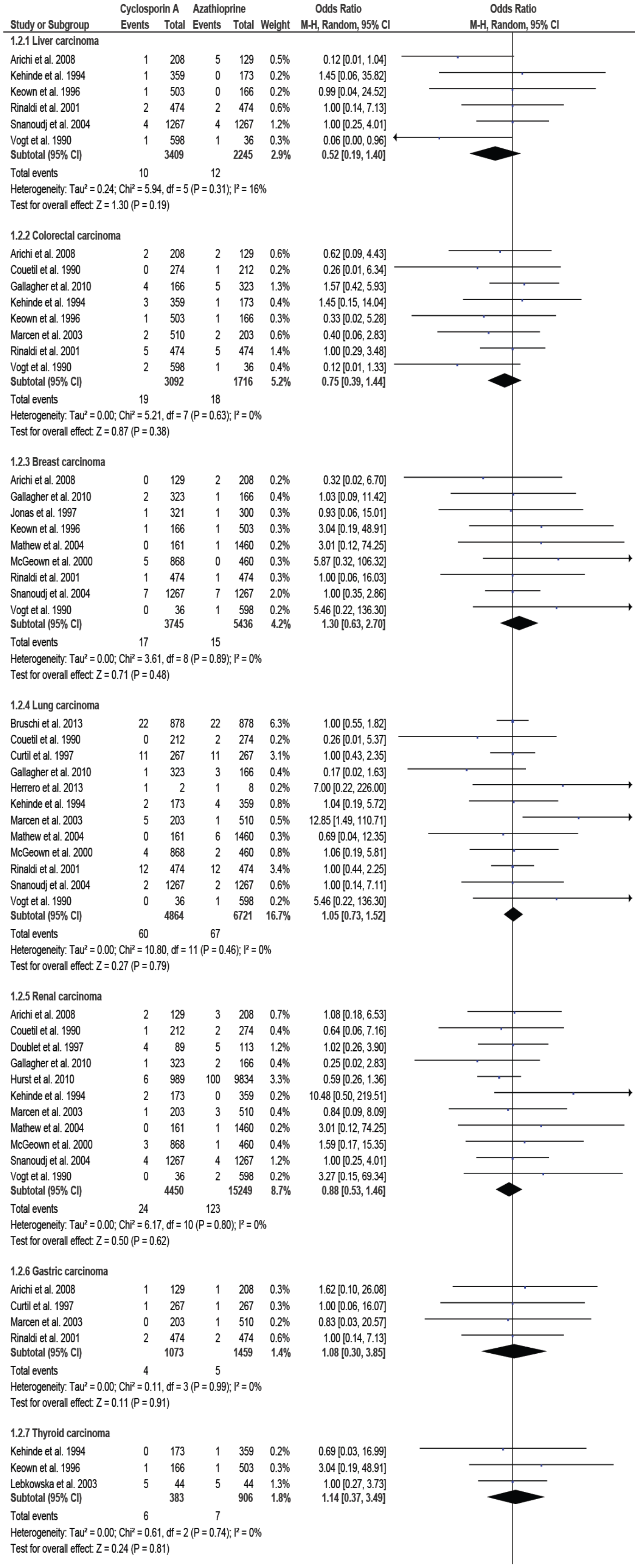

Figure S1. Cyclosporine A versus Tacrolimus.

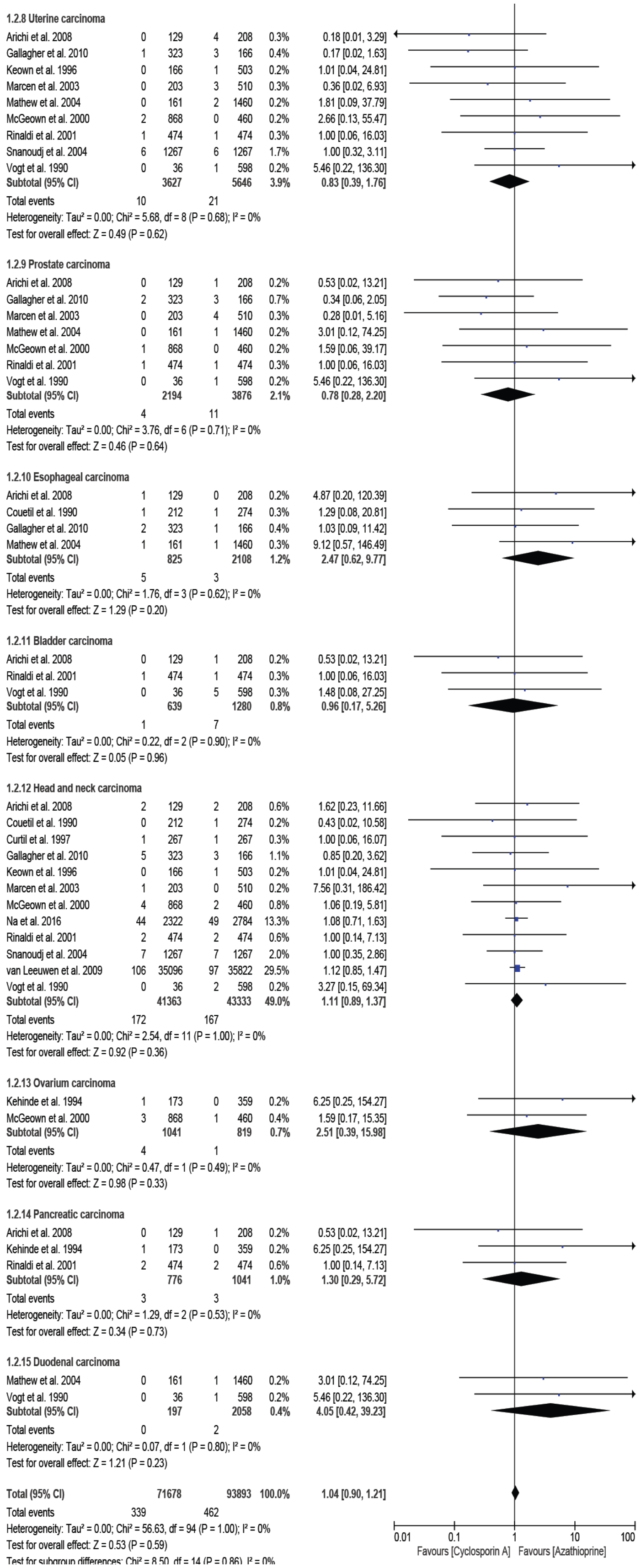
1.2.8 Uterine carcinoma
Arichi et al. 2008 0 129 4 208 0.3% 0.18 [0.01, 3.29]
Gallagher et al. 2010 1 323 3 166 0.4% 0.17 [0.02, 1.63]
Keown et al. 1996 0 166 1 503 0.2% 1.01 [0.04, 24.81]
Marcen et al. 2003 0 203 3 510 0.3% 0.36 [0.02, 6.93]
Mathew et al. 2004 0 161 2 1460 0.2% 1.81 [0.09, 37.79]
McGeown et al. 2000 2 868 0 460 0.2% 2.66 [0.13, 55.47]
Rinaldi et al. 2001 1 474 1 474 0.3% 1.00 [0.06, 16.03]
Snanoudj et al. 2004 6 1267 6 1267 1.7% 1.00 [0.32, 3.11]
Vogt et al. 1990 0 36 1 598 0.2% 5.46 [0.22, 136.30]
Subtotal (95% CI) 3627 5646 3.9% 0.83 [0.39, 1.76]
Total events 10 21
Heterogeneity: Tau² = 0.00; Chi² = 5.68, df = 8 (P = 0.68); I² = 0%
Test for overall effect: Z = 0.49 (P = 0.62)
1.2.9 Prostate carcinoma
Arichi et al. 2008 0 129 1 208 0.2% 0.53 [0.02, 13.21]
Gallagher et al. 2010 2 323 3 166 0.7% 0.34 [0.06, 2.05]
Marcen et al. 2003 0 203 4 510 0.3% 0.28 [0.01, 5.16]
Mathew et al. 2004 0 161 1 1460 0.2% 3.01 [0.12, 74.25]
McGeown et al. 2000 1 868 0 460 0.2% 1.59 [0.06, 39.17]
Rinaldi et al. 2001 1 474 1 474 0.3% 1.00 [0.06, 16.03]
Vogt et al. 1990 0 36 1 598 0.2% 5.46 [0.22, 136.30]
Subtotal (95% CI) 2194 3876 2.1% 0.78 [0.28, 2.20]
Total events 4 11
Heterogeneity: Tau² = 0.00; Chi² = 3.76, df = 6 (P = 0.71); I² = 0%
Test for overall effect: Z = 0.46 (P = 0.64)
1.2.10 Esophageal carcinoma
Arichi et al. 2008 1 129 0 208 0.2% 4.87 [0.20, 120.39]
Couetil et al. 1990 1 212 1 274 0.3% 1.29 [0.08, 20.81]
Gallagher et al. 2010 2 323 1 166 0.4% 1.03 [0.09, 11.42]
Mathew et al. 2004 1 161 1 1460 0.3% 9.12 [0.57, 146.49]
Subtotal (95% CI) 825 2108 1.2% 2.47 [0.62, 9.77]
Total events 5 3
Heterogeneity: Tau² = 0.00; Chi² = 1.76, df = 3 (P = 0.62); I² = 0%
Test for overall effect: Z = 1.29 (P = 0.20)
1.2.11 Bladder carcinoma
Arichi et al. 2008 0 129 1 208 0.2% 0.53 [0.02, 13.21]
Rinaldi et al. 2001 1 474 1 474 0.3% 1.00 [0.06, 16.03]
Vogt et al. 1990 0 36 5 598 0.3% 1.48 [0.08, 27.25]
Subtotal (95% CI) 639 1280 0.8% 0.96 [0.17, 5.26]
Total events 1 7
Heterogeneity: Tau² = 0.00; Chi² = 0.22, df = 2 (P = 0.90); I² = 0%
Test for overall effect: Z = 0.05 (P = 0.96)
1.2.12 Head and neck carcinoma
Arichi et al. 2008 2 129 2 208 0.6% 1.62 [0.23, 11.66]
Couetil et al. 1990 0 212 1 274 0.2% 0.43 [0.02, 10.58]
Curtil et al. 1997 1 267 1 267 0.3% 1.00 [0.06, 16.07]
Gallagher et al. 2010 5 323 3 166 1.1% 0.85 [0.20, 3.62]
Keown et al. 1996 0 166 1 503 0.2% 1.01 [0.04, 24.81]
Marcen et al. 2003 1 203 0 510 0.2% 7.56 [0.31, 186.42]
McGeown et al. 2000 4 868 2 460 0.8% 1.06 [0.19, 5.81]
Na et al. 2016 44 2322 49 2784 13.3% 1.08 [0.71, 1.63]
Rinaldi et al. 2001 2 474 2 474 0.6% 1.00 [0.14, 7.13]
Snanoudj et al. 2004 7 1267 7 1267 2.0% 1.00 [0.35, 2.86]
van Leeuwen et al. 2009 106 35096 97 35822 29.5% 1.12 [0.85, 1.47]
Vogt et al. 1990 0 36 2 598 0.2% 3.27 [0.15, 69.34]
Subtotal (95% CI) 41363 43333 49.0% 1.11 [0.89, 1.37]
Total events 172 167
Heterogeneity: Tau² = 0.00; Chi² = 2.54, df = 11 (P = 1.00); I² = 0%
Test for overall effect: Z = 0.92 (P = 0.36)
1.2.13 Ovarium carcinoma
Kehinde et al. 1994 1 173 0 359 0.2% 6.25 [0.25, 154.27]
McGeown et al. 2000 3 868 1 460 0.4% 1.59 [0.17, 15.35]
Subtotal (95% CI) 1041 819 0.7% 2.51 [0.39, 15.98]
Total events 4 1
Heterogeneity: Tau² = 0.00; Chi² = 0.47, df = 1 (P = 0.49); I² = 0%
Test for overall effect: Z = 0.98 (P = 0.33)
1.2.14 Pancreatic carcinoma
Arichi et al. 2008 0 129 1 208 0.2% 0.53 [0.02, 13.21]
Kehinde et al. 1994 1 173 0 359 0.2% 6.25 [0.25, 154.27]
Rinaldi et al. 2001 2 474 2 474 0.6% 1.00 [0.14, 7.13]
Subtotal (95% CI) 776 1041 1.0% 1.30 [0.29, 5.72]
Total events 3 3
Heterogeneity: Tau² = 0.00; Chi² = 1.29, df = 2 (P = 0.53); I² = 0%
Test for overall effect: Z = 0.34 (P = 0.73)
1.2.15 Duodenal carcinoma
Mathew et al. 2004 0 161 1 1460 0.2% 3.01 [0.12, 74.25]
Vogt et al. 1990 0 36 1 598 0.2% 5.46 [0.22, 136.30]
Subtotal (95% CI) 197 2058 0.4% 4.05 [0.42, 39.23]
Total events 0 2
Heterogeneity: Tau² = 0.00; Chi² = 0.07, df = 1 (P = 0.80); I² = 0%
Test for overall effect: Z = 1.21 (P = 0.23)
Total (95% CI) 71678 93893 100.0% 1.04 [0.90, 1.21]
Total events 339 462
Heterogeneity: Tau² = 0.00; Chi² = 56.63, df = 94 (P = 1.00); I² = 0%
Test for overall effect: Z = 0.53 (P = 0.59)
Test for subgroup differences: Chi² = 8.50, df = 14 (P = 0.86), I² = 0%
0.01 0.1 1 10 100
Favours [Cyclosporin A] Favours [Azathioprine]

Figure S2. Cyclosporine A versus Azathioprine.

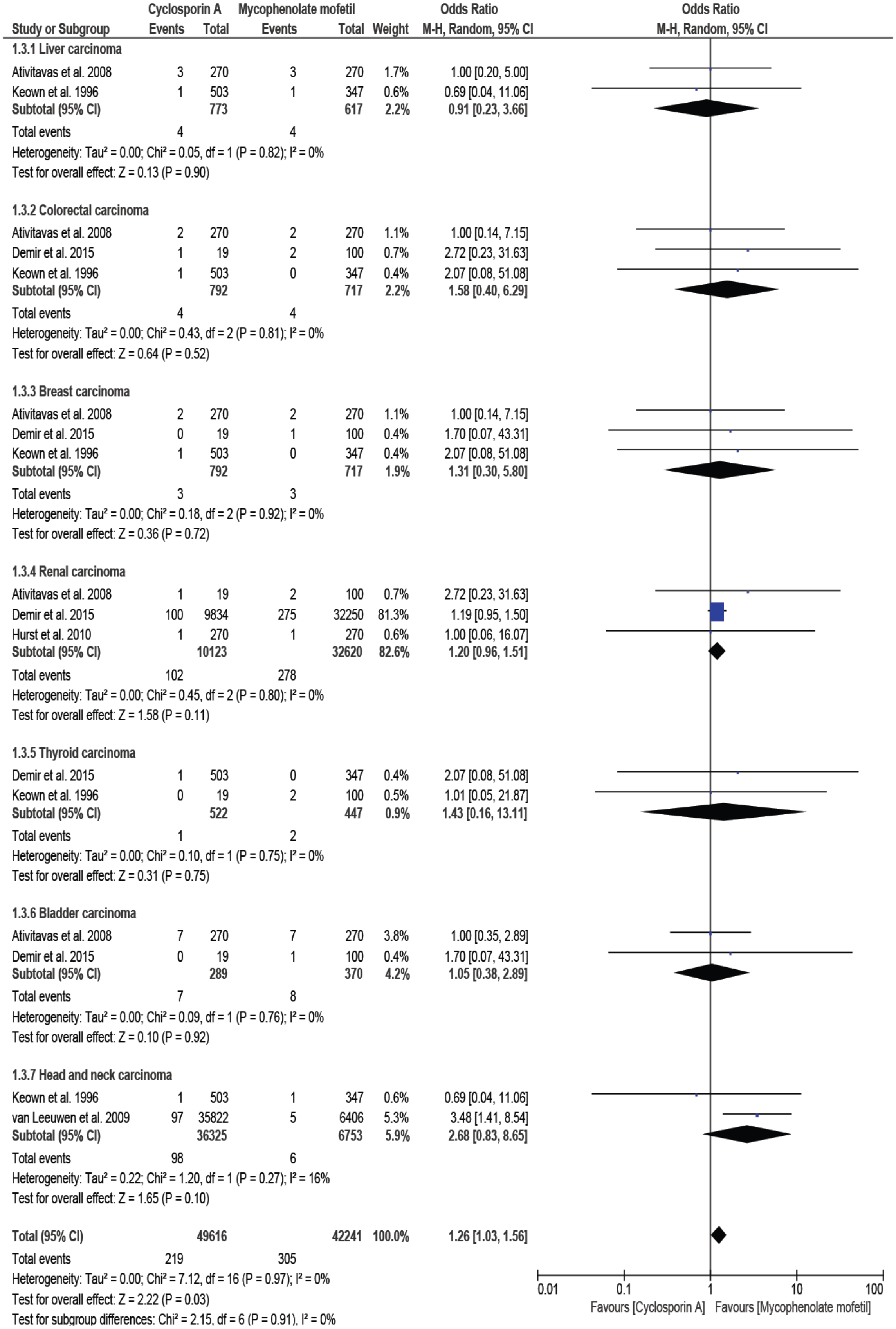

Figure S3. Cyclosporine A versus Mocyphenolate mofetil.

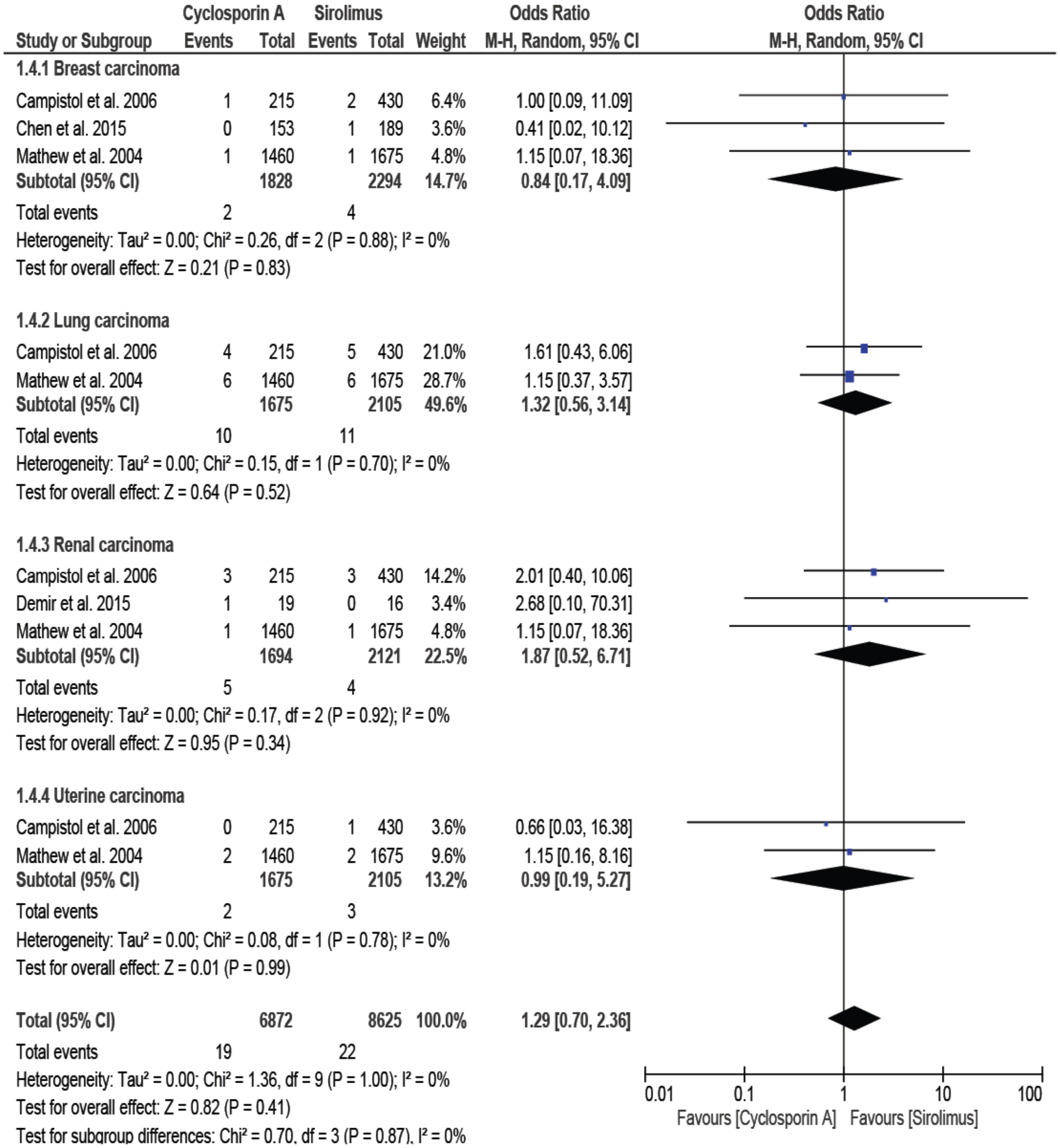

Figure S4. Cyclosporine A versus Sirolimus.

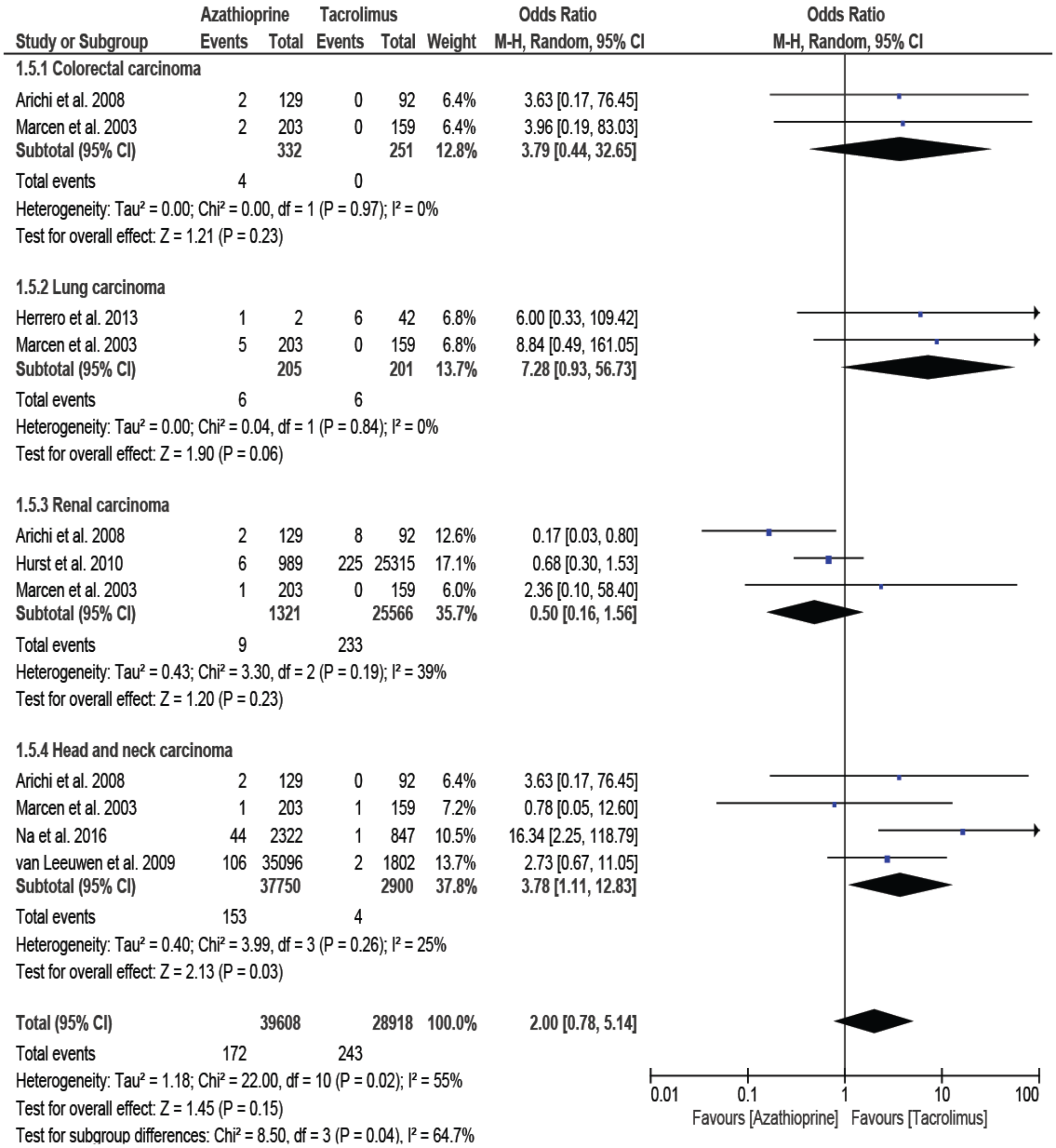

Figure S5. Azathioprine versus Tacrolimus.

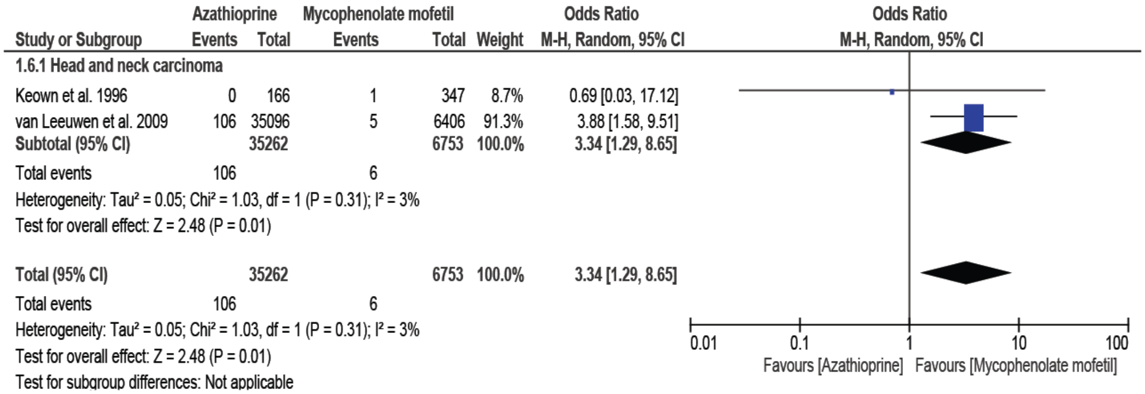

Figure S6. Azathioprine versus Mycophenolate mofetil.

Study or Subgroup	Mycophenolate mofetil Events	Mycophenolate mofetil Total	Tacrolimus Events	Tacrolimus Total	Weight	Odds Ratio M-H, Random, 95% CI
**1.7.1 Colorectal carcinoma**						
Albright et al. 2010	5	402	5	402	4.1%	1.00 [0.29, 3.48]
Demir et al. 2015	2	100	1	65	1.1%	1.31 [0.12, 14.70]
Fox et al. 2017	3	412	3	412	2.5%	1.00 [0.20, 4.98]
Lichtenberg et al. 2017	2	718	2	718	1.6%	1.00 [0.14, 7.12]
Tiwari et al. 2020	1	2100	1	2100	0.8%	1.00 [0.06, 16.00]
Yu et al. 2014	0	569	1	569	0.6%	0.33 [0.01, 8.19]
**Subtotal (95% CI)**		**4301**		**4266**	**10.7%**	**0.96 [0.45, 2.08]**
Total events	13		13			
Heterogeneity: Tau² = 0.00; Chi² = 0.49, df = 5 (P = 0.99); I² = 0%						
Test for overall effect: Z = 0.09 (P = 0.93)						
**1.7.2 Breast carcinoma**						
Albright et al. 2010	3	402	3	402	2.5%	1.00 [0.20, 4.98]
Demir et al. 2015	1	100	1	65	0.8%	0.65 [0.04, 10.52]
Fox et al. 2017	2	412	2	412	1.6%	1.00 [0.14, 7.13]
**Subtotal (95% CI)**		**914**		**879**	**4.9%**	**0.93 [0.30, 2.90]**
Total events	6		6			
Heterogeneity: Tau² = 0.00; Chi² = 0.08, df = 2 (P = 0.96); I² = 0%						
Test for overall effect: Z = 0.12 (P = 0.90)						
**1.7.3 Lung carcinoma**						
Albright et al. 2010	3	402	3	402	2.5%	1.00 [0.20, 4.98]
Fox et al. 2017	21	412	21	412	16.4%	1.00 [0.54, 1.86]
Herrero et al. 2013	2	31	6	42	2.3%	0.41 [0.08, 2.21]
Lichtenberg et al. 2017	3	718	3	718	2.5%	1.00 [0.20, 4.97]
Tiwari et al. 2020	4	2100	4	2100	3.3%	1.00 [0.25, 4.00]
Yu et al. 2014	0	569	2	569	0.7%	0.20 [0.01, 4.16]
**Subtotal (95% CI)**		**4232**		**4243**	**27.5%**	**0.89 [0.55, 1.44]**
Total events	33		39			
Heterogeneity: Tau² = 0.00; Chi² = 1.95, df = 5 (P = 0.86); I² = 0%						
Test for overall effect: Z = 0.46 (P = 0.65)						
**1.7.4 Renal carcinoma**						
Albright et al. 2010	3	402	3	402	2.5%	1.00 [0.20, 4.98]
Fox et al. 2017	21	412	21	412	16.4%	1.00 [0.54, 1.86]
Herrero et al. 2013	2	31	6	42	2.3%	0.41 [0.08, 2.21]
Lichtenberg et al. 2017	3	718	3	718	2.5%	1.00 [0.20, 4.97]
Yu et al. 2014	0	569	2	569	0.7%	0.20 [0.01, 4.16]
**Subtotal (95% CI)**		**2132**		**2143**	**24.3%**	**0.88 [0.53, 1.47]**
Total events	29		35			
Heterogeneity: Tau² = 0.00; Chi² = 1.92, df = 4 (P = 0.75); I² = 0%						
Test for overall effect: Z = 0.49 (P = 0.62)						
**1.7.5 Gastric carcinoma**						
Albright et al. 2010	1	402	1	402	0.8%	1.00 [0.06, 16.04]
Fox et al. 2017	1	412	1	412	0.8%	1.00 [0.06, 16.04]
Tiwari et al. 2020	1	2100	1	2100	0.8%	1.00 [0.06, 16.00]
Yu et al. 2014	1	569	2	569	1.1%	0.50 [0.05, 5.52]
**Subtotal (95% CI)**		**3483**		**3483**	**3.6%**	**0.81 [0.21, 3.06]**
Total events	4		5			
Heterogeneity: Tau² = 0.00; Chi² = 0.22, df = 3 (P = 0.97); I² = 0%						
Test for overall effect: Z = 0.31 (P = 0.75)						
**1.7.6 Thyroid carcinoma**						
Demir et al. 2015	2	100	2	65	1.6%	0.64 [0.09, 4.68]
Fox et al. 2017	1	412	1	412	0.8%	1.00 [0.06, 16.04]
Lichtenberg et al. 2017	1	718	1	718	0.8%	1.00 [0.06, 16.02]
**Subtotal (95% CI)**		**1230**		**1195**	**3.2%**	**0.80 [0.20, 3.25]**
Total events	4		4			
Heterogeneity: Tau² = 0.00; Chi² = 0.10, df = 2 (P = 0.95); I² = 0%						
Test for overall effect: Z = 0.31 (P = 0.76)						

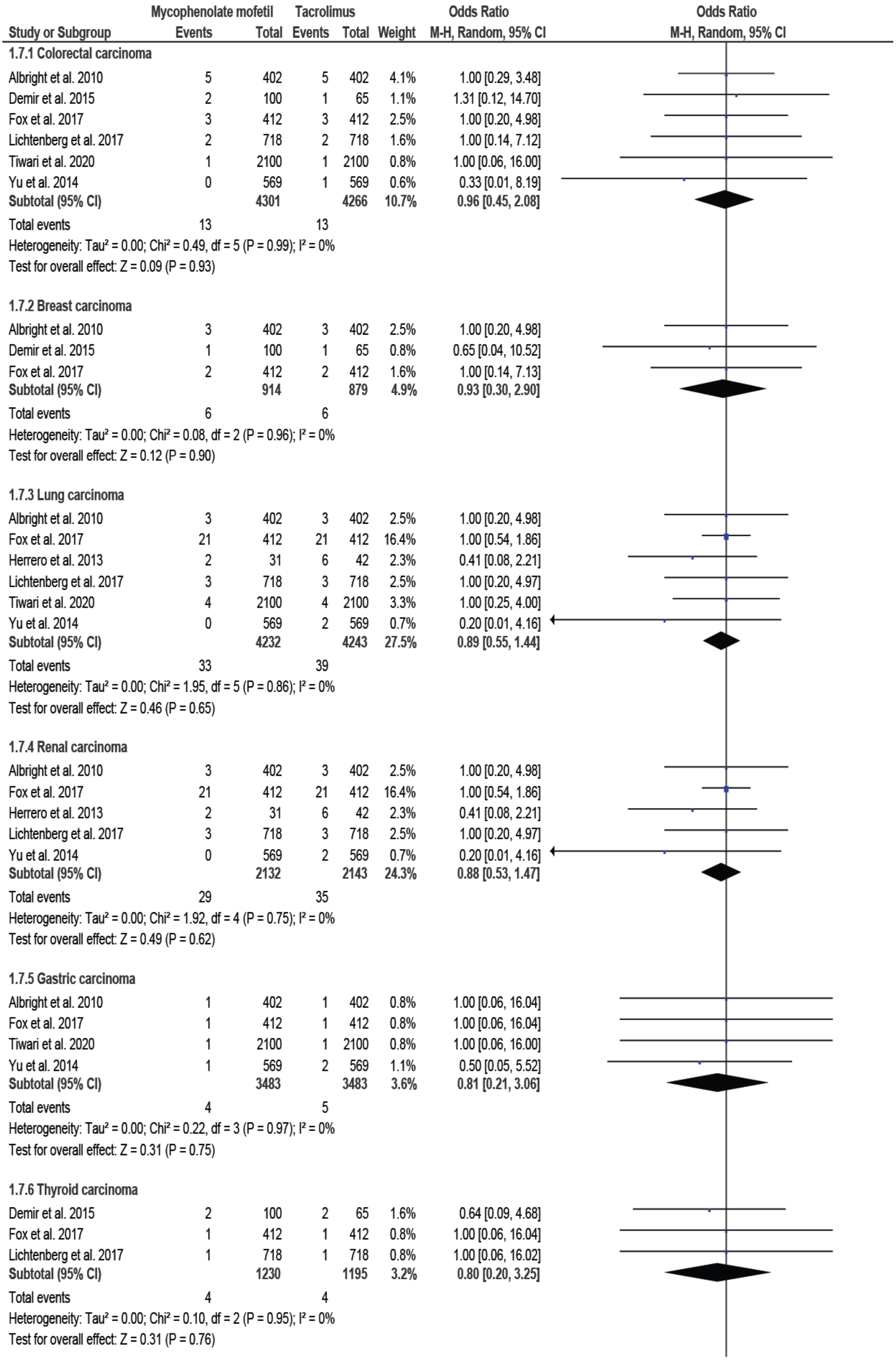

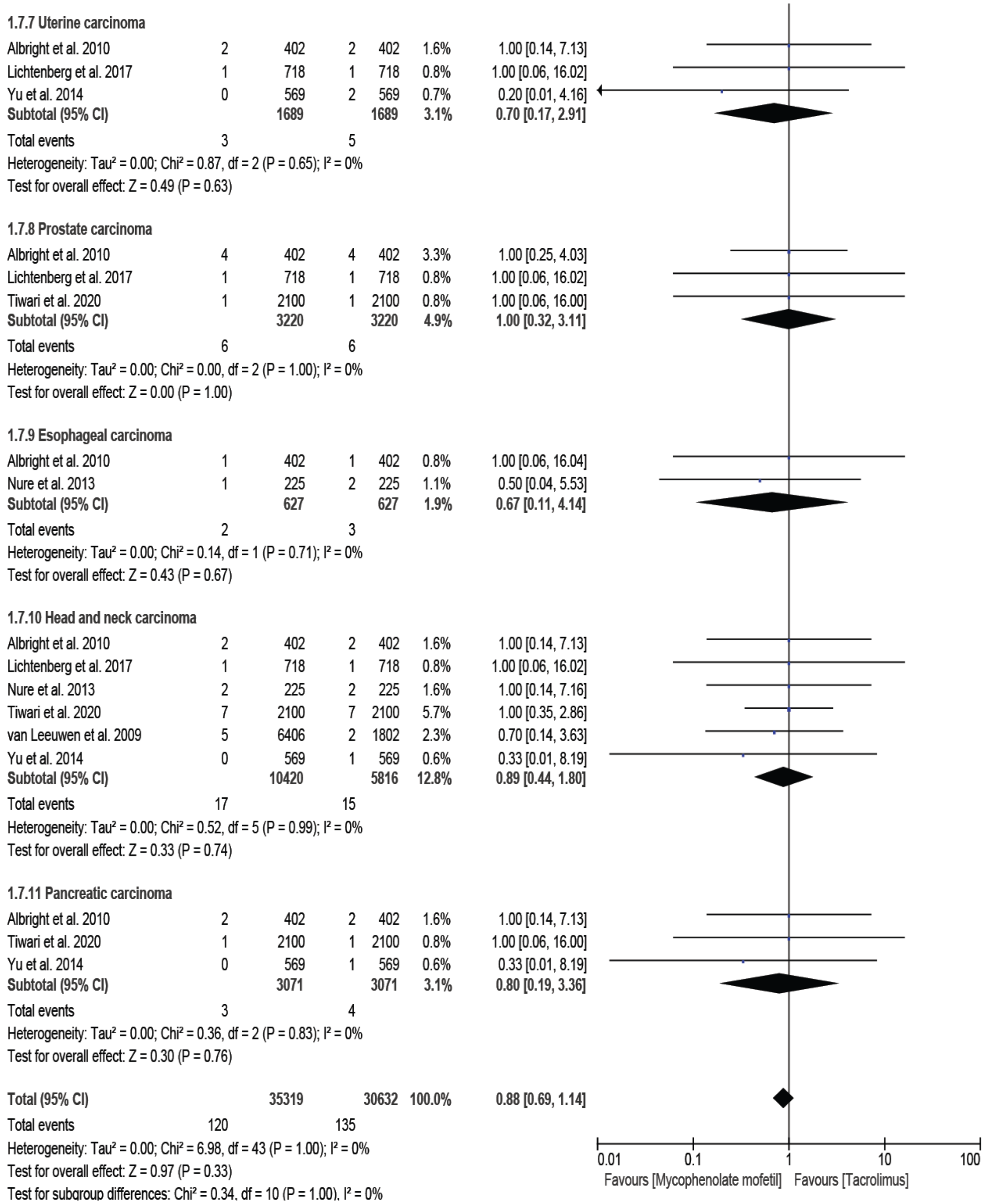

Figure S7. Mycophenolate mofetil versus Tacrolimus.

References

1. Aigner, F.; Boeckle, E.; Albright, J.; Kilo, J.; Boesmueller, C.; Conrad, F.; Wiesmayr, S.; Antretter, H.; Margreiter, R.; Mark, W.; et al. Malignancies of the colorectum and anus in solid organ recipients. *Transplant. Int.* **2007**, *20*, 497–504.
2. Albright, J.B.; Bonatti, H.; Stauffer, J.; Dickson, R.C.; Nguyen, J.; Harnois, D.; Jeanpierre, C.; Hinder, R.; Steers, J.; Chua, H.; et al. Colorectal and anal neoplasms following liver transplantation. *Colorectal Dis.* **2010**, *12*, 657–666.
3. Arichi, N.; Kishikawa, H.; Nishimura, K.; Mitsui, Y.; Namba, Y.; Tokugawa, S.; Ichikawa, Y. Malignancy following kidney transplantation. *Transplant. Proc.* **2008**, *40*, 2400–2402.
4. Ativitavas, T.; Jirasiritham, S.; Ngorsakun, P.; Pipatpannawong, K.; Mavichak, V. Malignancies in Renal Transplant Patients: 15 Years Experience in Thailand. *Transplant. Proc.* **2008**, *40*, 2403–2404.

5. Baccarani, U.; Piselli, P.; Serraino, D.; Adani, G.L.; Lorenzin, D.; Gambato, M.; Buda, A.; Zanus, G.; Vitale, A.; De Paoli, A.; et al. Comparison of de novo tumours after liver transplantation with incidence rates from Italian cancer registries. *Dig. Liver Dis.* **2010**, *42*, 55–60.
6. Bruschi, G.; Colombo, T.; Oliva, F.; Botta, L.; Morici, N.; Cannata, A.; Vittori, C.; Turazza, F.; Garascia, A.; Pedrazzini, G.; et al. Heart transplantation: 25 years' single-centre experience. *J. Cardiovasc. Med.* **2013**, *14*, 637–647.
7. Lee, J.U.; Kim, L.K.; Choi, J.M. Revisiting the Concept of Targeting NFAT to Control T Cell Immunity and Autoimmune Diseases. *Front. Immunol.* **2018**, *9*, 2747, doi:10.3389/fimmu.2018.02747.
8. Carenco, C.; Faure, S.; Herrero, A.; Assenat, E.; Duny, Y.; Danan, G.; Bismuth, M.; Chanques, G.; Ursic-Bedoya, J.; Jaber, S.; et al. Incidence of solid organ cancers after liver transplantation: Comparison with regional cancer incidence rates and risk factors. *Liver Int.* **2015**, *35*, 1748–1755.
9. Chambade, D.; Meria, P.; Tariel, E.; Verine, J.; De Kerviler, E.; Peraldi, M.N.; Glotz, D.; Desgrandchamps, F.; Mongiat-Artus, P. Nephron sparing surgery is a feasible and efficient treatment of T1a renal cell carcinoma in kidney transplant: A prospective series from a single center. *J. Urol.* **2008**, *180*, 2106–2109.
10. Chang, Y.L.; Lee, H.C.; Luo, H.L.; Chen, Y.T.; Chiang, P.H.; Cheng, Y.T. Preventive Role of mTOR Inhibitor in Post-Kidney Transplant Urothelial Carcinoma. *Transplant. Proc.* **2019**, *51*, 2731–2734.
11. Chen, J.L.; Ho, G.J.; Yang, Y.C.; Shih, M.H.; Chou, K.C.; Lee, M.C. High Incidence of Transitional Cell Carcinoma in Kidney Transplant Recipients in Eastern Taiwan. *Tzu Chi Med. J.* **2009**, *21*, 118–122.
12. Chen, K.H.; Lee, C.Y.; Wu, F.L.; Yang, C.Y.; Yeh, C.C.; Hu, R.H.; Tsai, M.K. De novo cancer avoidance after renal transplantation: A case-control study on low-dose sirolimus combined with a calcineurin inhibitor. *J. Formos. Med. Assoc.* **2015**, *114*, 526–531.
13. Couetil, J.P.; McGoldrick, J.P.; Wallwork, J.; English, T.A. Malignant tumors after heart transplantation. *J. Heart Transplant.* **1990**, *9*, 622–626.
14. Cox, J.; Colli, J.L. Urothelial cancers after renal transplantation. *Int. Urol. Nephrol.* **2011**, *43*, 681–686.
15. Curtil, A.; Robin, J.; Tronc, F.; Ninet, J.; Boissonnat, P.; Champsaur, G. Malignant neoplasms following cardiac transplantation. *Eur. J. Cardiothorac. Surg.* **1997**, *12*, 101–106.
16. Davis, N.F.; McLoughlin, L.C.; Dowling, C.; Power, R.; Mohan, P.; Hickey, D.; Smyth, G.; Eng, M.; Little, D.M. Incidence and long-term outcomes of squamous cell bladder cancer after deceased donor renal transplantation. *Clin. Transplant.* **2013**, *27*, E665–E668.
17. Demir, T.; Ozel, L.; Gokce, A.M.; Ata, P.; Kara, M.; Eris, C.; Ozdemir, E.; Titiz, M.I. Cancer Screening of Renal Transplant Patients Undergoing Long-Term Immunosuppressive Therapy. *Transplant. Proc.* **2015**, *47*, 1413–1417.
18. Doublet, J.D.; Peraldi, M.N.; Gattegno, B.; Thibault, P.; Sraer, J.D. Renal cell carcinoma of native kidneys: Prospective study of 129 renal transplant patients. *J. Urol.* **1997**, *158*, 42–44.
19. Eccher, A.; Boschiero, L.; Delahunt, B.; Cima, L.; Fior, F.; Nacchia, F.; Rost; , M.; Carraro, A.; Tedeschi, U.; et al. De novo renal neoplasia after kidney transplantation according to new 2016 WHO classification of renal tumors. *Ann. Transplant.* **2016**, *21*, 745–754.
20. Egeli, T.; Unek, T.; Ozbilgin, M.; Agalar, C.; Derici, S.; Akarsu, M.; Unek, I.T.; Aysin, M.; Bacakoglu, A.; Astarcioglu, I. De Novo Malignancies After Liver Transplantation: A Single Institution Experience. *Exp. Clin. Transplant.* **2017**, doi:10.6002/ect.2017.0111.
21. Elkentaoui, H.; Robert, G.; Pasticier, G.; Bernhard, J.C.; Couzi, L.; Merville, P.; Ravaud, A.; Ballanger, P.; Ferrière, J.M.; Waller; et al. Therapeutic Management of De Novo Urological Malignancy in Renal Transplant Recipients: The Experience of the French Department of Urology and Kidney Transplantation from Bordeaux. *Urology* **2010**, *75*, 126–132.
22. Filocamo, M.T.; Zanazzi, M.; Li Marzi, V.; Guidoni, L.; Villari, D.; Dattolo, E.; Nicita, G. Renal Cell Carcinoma of Native Kidney After Renal Transplantation: Clinical Relevance of Early Detection. *Transplant. Proc.* **2009**, *41*, 4197–4201.
23. Fox, B.D.; Ashquar, F.; Raviv, Y.; Rozengarten, D.; Straichman, O.; Izhakian, S.; Kramer, M.R. Tacrolimus Levels Are Not Associated with Risk of Malignancy in Lung Transplant Recipients. *Ann. Transplant.* **2017**, *22*, 677–681.
24. Frezza, E.E.; Fung, J.J.; van Thiel, D.H. Non-lymphoid cancer after liver transplantation. *Hepatogastroenterology* **1997**, *44*, 1172–1181.
25. Gallagher, M.P.; Kelly, P.J.; Jardine, M.; Perkovic, V.; Cass, A.; Craig, J.C.; Eris, J.; Webster, A.C. Long-term cancer risk of immunosuppressive regimens after kidney transplantation. *J. Am. Soc. Nephrol.* **2010**, *21*, 852–858.
26. Heinz-Peer, G.; Schoder, M.; , T.; Mayer, G.; Mostbeck, G.H. Prevalence of acquired cystic kidney disease and tumors in native kidneys of renal transplant recipients: A prospective US study. *Radiology* **1995**, *195*, 667–671.
27. Hurst, F.P.; Jindal, R.M.; Graham, L.J.; Falta, E.M.; Elster, E.A.; Stackhouse, G.B.; Agodoa, L.Y.; Lentine, K.L.; Salifu, M.O.; Abbott, K.C. Incidence, predictors, costs, and outcome of renal cell carcinoma after kidney transplantation: USRDS experience. *Transplantation* **2010**, *90*, 898–904.
28. Jonas, S.; Rayes, N.; Neumann, U.; Neuhaus, R.; Bechstein, W.O.; Guckelberger, O.; Tullius, S.G.; Serke, S.; Neuhaus, P. De novo malignancies after liver transplantation using tacrolimus-based protocols or cyclosporine-based quadruple immunosuppression with an interleukin-2 receptor antibody or antithymocyte globulin. *Cancer* **1997**, *80*, 1141–1150.
29. Kahan, B.D.; Yakupoglu, Y.K.; Schoenberg, L.; Knight, R.J.; Katz, S.M.; Lai, D.; Van Buren, C.T. Low incidence of malignancy among sirolimus/cyclosporine-treated renal transplant recipients. *Transplantation* **2005**, *80*, 749–758.
30. Kamal, M.M.; Soliman, S.M.; Shokeir, A.A.; Abol-Enein, H.; Ghoneim, M.A. Bladder carcinoma among live-donor renal transplant recipients: A single-centre experience and a review of the literature. *BJU Int.* **2008**, *101*, 30–35.

31. Kanaan, N.; Raggi, C.; Goffin, E.; De Meyer, M.; Mourad, M.; Jadoul, M.; Beguin, C.; Kabamba, B.; Borbath, I.; Pirson, Y.; et al. Outcome of hepatitis B and C virus-associated hepatocellular carcinoma occurring after renal transplantation. *J. Viral Hepat.* **2017**, *24*, 430–435.
32. Karczewski, M.; Czapiewski, W.; Karczewski, J. Urologic de novo malignancies after kidney transplantation: A single center experience. *Transplant. Proc.* **2012**, *44*, 1293–1297.
33. Kauffman, H.M.; Cherikh, W.S.; Cheng, Y.; Hanto, D.W.; Kahan, B.D. Maintenance immunosuppression with target-of-rapamycin inhibitors is associated with a reduced incidence of de novo malignancies. *Transplantation* **2005**, *80*, 883–889.
34. Kehinde, E.O.; Petermann, A.; Morgan, J.D.; Butt, Z.A.; Donnelly, P.K.; Veitch, P.S.; Bell, P.R. Triple therapy and incidence of de novo cancer in renal transplant recipients. *Br. J. Surg.* **1994**, *81*, 985–986.
35. Kellerman, L.; Neugut, A.; Burke, B.; Mancini, D. Comparison of the incidence of de novo solid malignancies after heart transplantation to that in the general population. *Am. J. Cardiol.* **2009**, *103*, 562–566.
36. Mycophenolate mofetil for the prevention of acute rejection of primary cadaveric kidney transplants: Status of the MYC 1866 study at 1 year. The U.S. Mycophenolate Mofetil Study Group. *Transplant. Proc.* **1997**, *29*, 348–349, doi:10.1016/s0041-1345(96)00302-8.
37. Kim, D.Y.; Abouljoud, M.; Parasuraman, R. The role of microscopic hematuria in the evaluation of urologic malignancy in renal transplant recipients. *Transplant. Proc.* **2010**, *42*, 1641–1642.
38. Kim, H.J.; Lee, T.W.; Ihm, C.G.; Kim, M.J. Prevalence of cancers in Korean recipients of renal transplants. *Nephrology* **2002**, *7*, 198–204.
39. Kwak, H.Y.; Chae, B.J.; Bae, J.S.; Jung, S.S.; Song, B.J. Breast cancer after kidney transplantation: A single institution review. *World J. Surg. Oncol.* **2013**, *11*, 77.
40. Lebkowska, U.; Malyszko, J.S.; Malyszko, J.; Dzieciol, J.; Walecki, J.; Mysliwiec, M. Thyroid function and incidentalomas in kidney transplant recipients. *Med. Sci Monit* **2003**, *9*, MT8–MT11.
41. Li, H.Z.; Xia, M.; Han, Y.; Xu, X.G.; Zhang, Y.S. De novo urothelial carcinoma in kidney transplantation patients with end-stage aristolochic acid nephropathy in China. *Urol. Int.* **2009**, *83*, 200–205.
42. Lichtenberg, S.; Rahamimov, R.; Green, H.; Fox, B.D.; Mor, E.; Gafter, U.; Chagnac, A.; Rozen-Zvi, B. The incidence of post-transplant cancer among kidney transplant recipients is associated with the level of tacrolimus exposure during the first year after transplantation. *Eur. J. Clin. Pharm.* **2017**, *73*, 819–826.
43. Herrero, J.I. De novo malignancies following liver transplantation: Impact and recommendations. *Liver Transplant.* **2009**, *15* (Suppl. S2), S90–S94, doi:10.1002/lt.21898.
44. Lopez-Pintor, R.M.; Hern; ez, G.; de Arriba, L.; de Andres, A. Lip cancer in renal transplant patients. *Oral Oncol.* **2011**, *47*, 68–71.
45. Marcen, R.; Pascual, J.; Tato, A.M.; Teruel, J.L.; Villafruela, J.J.; Fern; ez, M.; Tenorio, M.; Burgos, F.J.; Ortuno, J. Influence of immunosuppression on the prevalence of cancer after kidney transplantation. *Transplant. Proc.* **2003**, *35*, 1714–1716.
46. Mathew, T.; Kreis, H.; Friend, P. Two-year incidence of malignancy in sirolimus-treated renal transplant recipients: Results from five multicenter studies. *Clin. Transplant.* **2004**, *18*, 446–449.
47. McGeown, M.G.; Douglas, J.F.; Middleton, D. One thousand renal transplants at Belfast City Hospital: Post-graft neoplasia 1968–1999, comparing azathioprine only with cyclosporin-based regimes in a single centre. *Clin. Transplant.* **2000**, *14*, 193–202.
48. Mizuno, S.; Hayasaki, A.; Ito, T.; Fujii, T.; Iizawa, Y.; Kato, H.; Murata, Y.; Tanemura, A.; Kuriyama, N.; Azumi, Y.; et al. De Novo Malignancy Following Adult-to-Adult Living Donor Liver Transplantation Focusing on Posttransplantation Lymphoproliferative Disorder. *Transplant. Proc.* **2018**, *50*, 2699–2704.
49. Na, R.; Laaksonen, M.A.; Grulich, A.E.; Meagher, N.S.; McCaughan, G.W.; Keogh, A.M.; Vajdic, C.M. High azathioprine dose and lip cancer risk in liver, heart, and lung transplant recipients: A population-based cohort study. *J. Am. Acad. Derm.* **2016**, *74*, 1144–1152.e1146.
50. Neuzillet, Y.; Lay, F.; Luccioni, A.; Daniel, L.; Berland, Y.; Coulange, C.; Lechevallier, E. De novo renal cell carcinoma of native kidney in renal transplant recipients. *Cancer* **2005**, *103*, 251–257.
51. Nure, E.; Frongillo, F.; Lirosi, M.C.; Grossi, U.; Sganga, G.; Avolio, A.W.; Siciliano, M.; Addolorato, G.; Mariano, G.; Agnes, S. Incidence of upper aerodigestive tract cancer after liver transplantation for alcoholic cirrhosis: A 10-year experience in an Italian center. *Transplant. Proc.* **2013**, *45*, 2733–2735.
52. Oezcelik, A.; Kaiser, G.M.; Dechene, A.; Treckmann, J.W.; Sotiropoulos, G.C.; Reinhardt, R.; Saner, F.H.; Paul, A. Progression to adenocarcinoma in Barrett's esophagus after liver transplantation. *Transplantation* **2011**, *91*, 1250–1253.
53. Opelz, G.; Unterrainer, C.; Susal, C.; Dohler, B. Immunosuppression with mammalian target of rapamycin inhibitor and incidence of post-transplant cancer in kidney transplant recipients. *Nephrol. Dial. Transplant.* **2016**, *31*, 1360–1367.
54. Palazzetti, A.; Bosio, A.; Dalmasso, E.; Destefanis, P.; Fop, F.; Pisano, F.; Segoloni, G.; Biancone, L.; Volpe, A.; Di Domenico, A.; et al. De Novo Bladder Urothelial Neoplasm in Renal Transplant Recipients: A Retrospective, Multicentered Study. *Urol. Int.* **2018**, *100*, 185–192.
55. Park, H.W.; Hwang, S.; Ahn, C.S.; Kim, K.H.; Moon, D.B.; Ha, T.Y.; Song, G.W.; Jung, D.H.; Park, G.C.; Namgoong, J.M.; et al. De novo malignancies after liver transplantation: Incidence comparison with the Korean cancer registry. *Transplant. Proc.* **2012**, *44*, 802–805.
56. Park, J.M.; Choi, M.G.; Yang, C.W.; Jung, C.K.; Lee, S.K.; Yoon, A.R.; Kim, Y.S.; Chung, I.S. Increased incidence of gastric cancer in renal transplant recipients. *J. Clin. Gastroenterol.* **2012**, *46*, e87–e91.

57. Park, M.J.; Roh, J.L.; Choi, S.H.; Nam, S.Y.; Kim, S.Y.; Lee, Y.S. De novo head and neck cancer arising in solid organ transplantation recipients: The Asan Medical Center experience. *Auris Nasus Larynx* **2018**, *45*, 838–845.
58. Ploussard, G.; Chambade, D.; Meria, P.; Gaudez, F.; Tariel, E.; Verine, J.; De Bazelaire, C.; Peraldi, M.N.; Glotz, D.; Desgr; et al. Biopsy-confirmed de novo renal cell carcinoma (RCC) in renal grafts: A single-centre management experience in a 2396 recipient cohort. *BJU Int.* **2012**, *109*, 195–199.
59. Raeisi, D.; Payandeh, M.; Madani, S.H.; Zare, M.E.; Kansestani, A.N.; Hashemian, A.H. Kaposi's sarcoma after kidney transplantation: A 21-years experience. *Int. J. Hematol. Oncol. Stem Cell Res.* **2013**, *7*, 29–33.
60. Rinaldi, M.; Pellegrini, C.; D'Armini, A.M.; Aiello, M.; Negri, M.; Arbustini, E.; Ippoliti, G.; Vigano, M. Neoplastic disease after heart transplantation: Single center experience. *Eur. J. Cardiothorac. Surg.* **2001**, *19*, 696–701.
61. Saigal, S.; Norris, S.; Muiesan, P.; Rela, M.; Heaton, N.; O'Grady, J. Evidence of differential risk for posttransplantation malignancy based on pretransplantation cause in patients undergoing liver transplantation. *Liver Transplant.* **2002**, *8*, 482–487.
62. Schmidt, R.; Stippel, D.; Krings, F.; Pollok, M. Malignancies of the genito-urinary system following renal transplantation. *Br. J. Urol.* **1995**, *75*, 572–577.
63. Shoji, F.; Toyokawa, G.; Harada, N.; Itoh, S.; Harimoto, N.; Ikegami, T.; Okamoto, T.; Soejima, Y.; Yoshizumi, T.; Maehara, Y. Surgical Treatment and Outcome of Patients with De Novo Lung Cancer After Liver Transplantation. *Anticancer Res.* **2017**, *37*, 2619–2623.
64. Singh, S.K.; Gupta, A.K.; Jha, V.; Kohli, H.S.; Gupta, K.L.; Minz, M.; Sakhuja, V. Treatment of oropharyngeal cancer in renal transplant recipients without cessation of immunosuppressive therapy. *Transplant. Proc.* **2006**, *38*, 2088–2089.
65. Slavis, S.A.; Novick, A.C.; Steinmuller, D.R.; Streem, S.B.; Braun, W.E.; Straffon, R.A.; Mastroianni, B.; Graneto, D. Outcome of renal transplantation in patients with a functioning graft for 20 years or more. *J. Urol.* **1990**, *144*, 20–22.
66. Snanoudj, R.; Kriaa, F.; Arzouk, N.; Beaudreuil, S.; Hiesse, C.; Durrbach, A.; Charpentier, B. Single-center experience with cyclosporine therapy for kidney transplantation: Analysis of a twenty-year period in 1200 patients. *Transplant. Proc.* **2004**, *36*, 83s–88s.
67. Sobieszczanska-Malek, M.; Komuda, K.; Piotrowska, M.; Korewicki, J.; Malek, G.; Leszek, P.; Zielinski, T. Incidence of malignancies in cardiac allograft recipients—A single-center experience. *Ann. Transplant.* **2013**, *18*, 88–94.
68. Stauch, C.; Fischer, B.; Bernhard, A. Malignancies after Heart Transplantation. *Oncol. Res. Treat.* **1993**, *16*, 338–343, doi:10.1159/000218286.
69. Sun, I.O.; Ko, Y.M.; Kim, E.Y.; Park, K.S.; Jung, H.S.; Ko, S.H.; Chung, B.H.; Choi, B.S.; Park, C.W.; Kim, Y.S.; et al. Clinical characteristics and outcomes in renal transplant recipients with renal cell carcinoma in the native kidney. *Korean J. Intern. Med.* **2013**, *28*, 347–351.
70. Tillou, X.; Doerfler, A.; Collon, S.; Kleinclauss, F.; Patard, J.J.; Badet, L.; Barrou, B.; Audet, M.; Bensadoun, H.; Berthoux, E.; et al. De novo kidney graft tumors: Results from a multicentric retrospective national study. *Am. J. Transplant.* **2012**, *12*, 3308–3315.
71. Tiwari, A.; Saigal, S.; Choudhary, N.S.; Saha, S.; Rastogi, A.; Bhangui, P.; Saraf, N.; Srinivasan, T.; Yadav, S.K.; Gautam, D.; et al. De Novo Malignancy After Living Donor Liver Transplantation: A Large Volume Experience. *J. Clin. Exp. Hepatol.* **2020**, *10*, 448–452.
72. Tomaszewski, J.J.; Larson, J.A.; Smaldone, M.C.; Hayn, M.H.; Jackman, S.V. Management of Bladder Cancer following Solid Organ Transplantation. *Adv. Urol.* **2011**, *2011*, 256985.
73. van Leeuwen, M.T.; Grulich, A.E.; McDonald, S.P.; McCredie, M.R.; Amin, J.; Stewart, J.H.; Webster, A.C.; Chapman, J.R.; Vajdic, C.M. Immunosuppression and other risk factors for lip cancer after kidney transplantation. *Cancer Epidemiol. Biomark. Prev.* **2009**, *18*, 561–569.
74. Vegso, G.; Toronyi, E.; Hajdu, M.; Piros, L.; Gorog, D.; Deak, P.A.; Doros, A.; Peter, A.; Langer, R.M. Renal cell carcinoma of the native kidney: A frequent tumor after kidney transplantation with favorable prognosis in case of early diagnosis. *Transplant. Proc.* **2011**, *43*, 1261–1263.
75. Vogt, P.; Frei, U.; Repp, H.; Bunzendahl, H.; Oldhafer, K.; Pichlmayr, R. Malignant tumours in renal transplant recipients receiving cyclosporin: Survey of 598 first-kidney transplantations. *Nephrol. Dial. Transplant.* **1990**, *5*, 282–288.
76. Wang, Y.J.; Chi, N.H.; Chou, N.K.; Huang, S.C.; Wang, C.H.; Wu, I.H.; Yu, H.Y.; Chen, Y.S.; Tsao, C.I.; Shun, C.T.; et al. Malignancy After Heart Transplantation Under Everolimus Versus Mycophenolate Mofetil Immunosuppression. *Transplant. Proc.* **2016**, *48*, 969–973.
77. Wu, B.; Wang, K.; Mo, C.B.; Shen, Z.Y. De novo malignancies in renal transplant recipients: Experience at a single center in China. *Int. J. Clin. Exp. Med.* **2015**, *8*, 2911–2916.
78. Yanik, E.L.; Gustafson, S.K.; Kasiske, B.L.; Israni, A.K.; Snyder, J.J.; Hess, G.P.; Engels, E.A.; Segev, D.L. Sirolimus use and cancer incidence among US kidney transplant recipients. *Am. J. Transplant.* **2015**, *15*, 129–136.
79. Yilmaz Akcay, E.; Tepeoglu, M.; Ozdemir, B.H.; Deniz, E.; Borcek, P.; Haberal, M. De Novo Malignant Neoplasms in Renal Transplant Patients. *Exp. Clin. Transplant.* **2016**, *14*, 100–105.
80. Yoshimura, N.; Akioka, K.; Ushigome, H.; Kadotani, Y.; Ogino, S.; Wakabayashi, Y.; Higuchi, A.; Nobori, S.; Kaihara, S.; Okamoto, M. Twenty-five-year survival of living related kidney transplants: Thirty-five years' experience. *Transplant. Proc.* **2005**, *37*, 687–689.
81. Yserbyt, J.; Verleden, G.M.; Dupont, L.J.; Van Raemdonck, D.E.; Dooms, C. Bronchial carcinoma after lung transplantation: A single-center experience. *J. Heart Lung Transplant.* **2012**, *31*, 585–590.

82. Yu, S.; Gao, F.; Yu, J.; Yan, S.; Wu, J.; Zhang, M.; Wang, W.; Zheng, S. De novo cancers following liver transplantation: A single center experience in China. *PLoS ONE* **2014**, *9*, doi:10.1371/journal.pone.0085651.
